# Clinical significance of Annexin A2 expression in oral squamous cell carcinoma and its influence on cell proliferation, migration and invasion

**DOI:** 10.1038/s41598-021-84675-y

**Published:** 2021-03-03

**Authors:** Yingyi Ma, Haiye Wang

**Affiliations:** 1 Department of Periodontal, Henan Provincial Stomatology Hospital, The First Affiliated Hospital of Zhengzhou University, Zhengzhou, 450052 Henan Province China; 2 Special Department, Henan Provincial Stomatology Hospital, The First Affiliated Hospital of Zhengzhou University, No. 1 Jianshe East Road, Zhengzhou, 450052 Henan Province China

**Keywords:** Cancer, Cell biology

## Abstract

Oral squamous cell carcinoma (OSCC) is the most common malignant epithelial neoplasm of the head and neck, with poorer prognosis. There is lack of specific targets for diagnosis and treatment of OSCC at present. Annexin A2 (ANXA2) is involved in cell angiogenesis, invasion, proliferation and metastasis. In this study, the significance and effect of ANXA2 on OSCC and OSCC cells were explored from the clinical and basic study. First, ANXA2 expression in OSCC tissues and adjacent non-cancer tissues of 124 patients were detected, and the correlation between ANXA2 expression and clinical parameters were analyzed. The results found that ANXA2 was highly expressed in OSCC tissues, and was associated with the TNM stage, tumor differentiation, lymph node metastasis and poor survival of OSCC patients. The expression of ANXA2 in OSCC cells were higher than the normal oral cells. And knockdown of ANXA2 by transfecting ANXA2-siRNA could suppress the proliferation, migration, and invasion abilities of OSCC cells. Overall, ANXA2 expression is correlated with poor survival of OSCC patients, and silencing of ANXA2 suppress the proliferation, migration and invasion of OSCC cells.

## Introduction

Oral squamous cell carcinoma (OSCC) is the world’s eighth malignancy and the most common malignant epithelial neoplasm of the head and neck^1^. In recent years, the incidence of OSCC has been on the rise and the onset age has been getting younger^2^. Though the diagnosis and treatment technology of OSCC have improved over the last three decades, the prognosis is still poor and there is still a lack of specific targets for diagnosis and treatment of OSCC at present^3^. Thus, the identification of biological markers related to the biological behaviors of OSCC cells, is crucial for the diagnosis, treatment and prognosis of OSCC.

Annexin A2 (ANXA2), a 36 kDa membrane protein on cell surface, is a member of calcium-dependent phospholipid-binding protein family, which is widely expressed in various eukaryotic cells. Previous researches have identified that ANXA2 is involved in cell angiogenesis, invasion, proliferation and metastasis^4–6^. Abnormal expression of ANXA2 has been verified to be associated with multiple malignancies, such as gastric carcinoma^7^, pancreatic cancer^8^, colorectal cancer^9^, breast cancer^10^, prostate cancer^11^ and esophagus cancer^12^. Furthermore, it is reported that ANXA2 plays an important role in biological behaviors involving cytoskeleton, cell phenotype and other changes in cell malignant transformation, tumor growth, tumor cell adhesion and metastasis^13^. However, the physiological functions of ANXA2 in OSCC is still unclear. In the present study, the correlation among ANXA2 expression, clinicopathological characteristics, and prognosis of OSCC patients were analyzed. The siRNA interference experiments were used to analyze the function of ANXA2 in modulating cell migration, invasion, and proliferation, to evaluate whether ANXA2 could be considered as a potential target of OSCC.

## Results

### Expression of ANXA2 in OSCC tissues and its correlation with clinicopathological features

**Figure 1:**
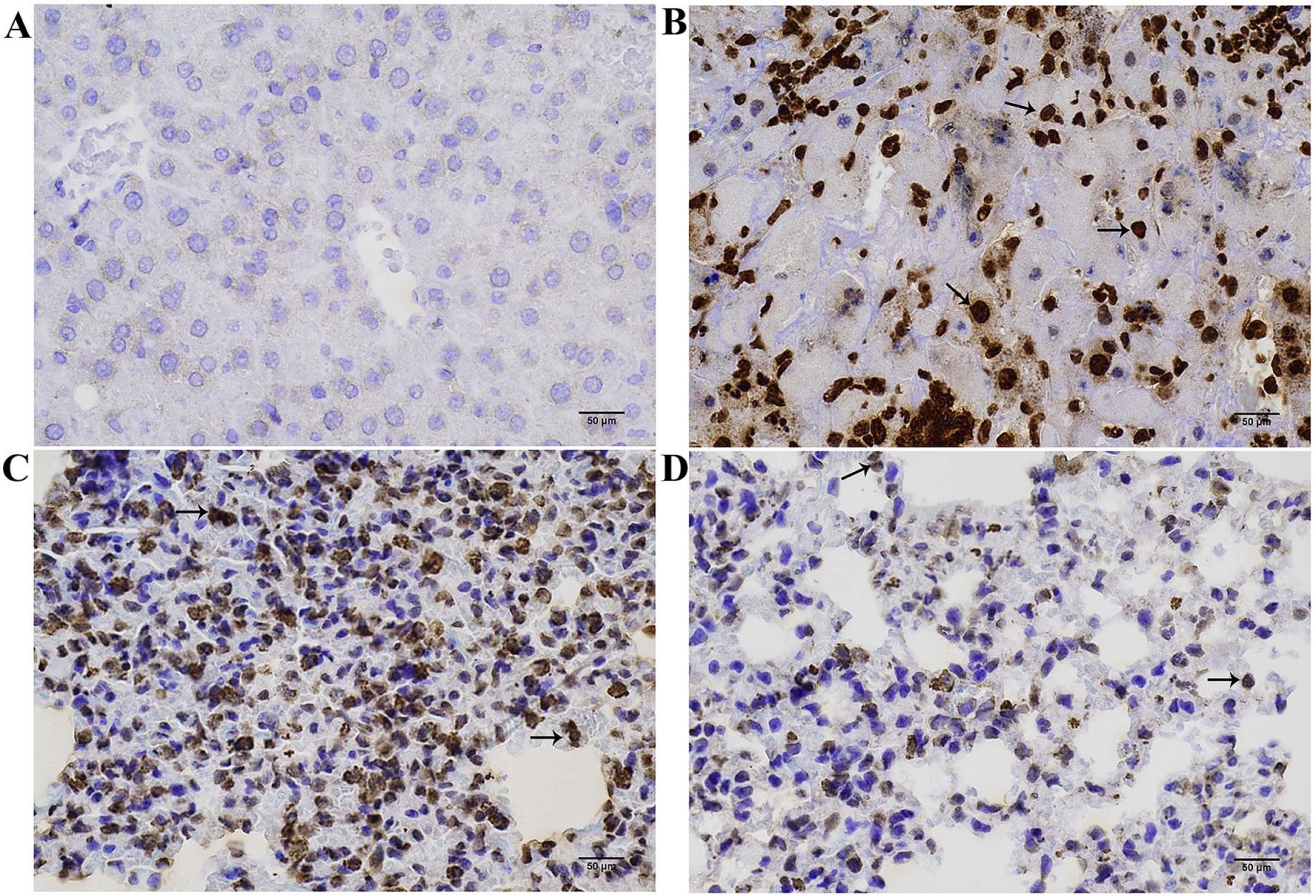
The typical immunohistochemical images of ANXA2 in adjacent non-cancer tissue and OSCC tissue. (**A**) The negative expression of ANXA2 in adjacent non-cancer tissue; (**B**) The positive expression of ANXA2 in OSCC tissue; (**C**) The strongly positive expression of ANXA2 in low differentiated OSCC tissue; (**D**) The weakly positive expression of ANXA2 in high differentiated OSCC tissue. The black arrowheads marked ANXA2 stain positive cells. Scale bar: 25 μm.

IHC analysis was used to detect the expression of ANXA2 in OSCC tissues (Fig. 1) and the results showed that the positive expression rate of ANXA2 in OSCC tissues was 77.4% (96/124), which was significantly higher than that in the normal oral epithelium tissues [24.2% (30/124)]. And the positive expression rate of ANXA2 in low differentiated OSCC tissues was higher than that in the high differentiated OSCC tissues (see the supplementary files). According to the ANXA2 expression, OSCC patients were divided into the positive ANXA2 group (n = 96) and negative ANXA2 group (n = 28). The clinical parameters between the positive and negative ANXA2 groups were compared (Table 1) and the results indicated that there were differences of TNM stage, tumor differentiation and lymph node metastasis between the two groups and ANXA2 expression may be correlated with these features(*P* < 0.05).

**Table 1:** Correlation of ANXA2 expression with clinicopathological features of OSCC patients.

Clinicopathological features	ANXA2 negative group (n = 28)	ANXA2 positive group (n = 96)	*F/χ*^*2*^	*P*
Age	52.32 ± 13.87	55.59 ± 12.64	1.179	0.241
Tumor size(cm)	3.18 ± 0.85	3.32 ± 0.95	0.740	0.460
**Gender**				
Male	18	50	1.303	0.254
Female	10	46		
**TNM stage**				
I + II	23	45	10.887	0.001
III + IV	5	51		
**Degree of differentiation**				
Low	7	26	11.053	0.004
Moderately	4	42		
High	17	28		
**Lymph node metastasis**				
No	26	69	5.326	0.021
Yes	2	27		
**Invasion depth**				
< 5 mm	25	71	2.913	0.088
≥ 5 mm	3	25		
**Tumor location**				
Tongue	8	30	2.668	0.263
Gingiva	16	40		
Others	4	26		

### Correlation of ANXA2 with the survival of OSCC patients

**Figure 2:**
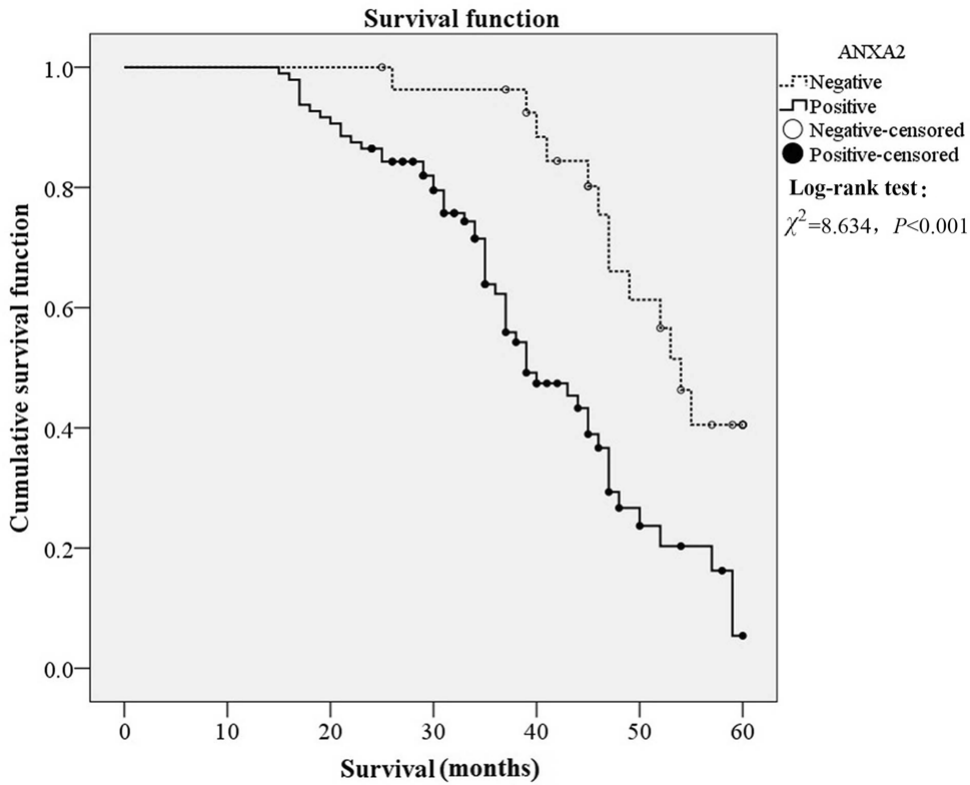
Kaplan–Meier analysis for the survival of OSCC patients with ANXA2 positive and negative expression. ANXA2 positive expression had a poor cumulative survival.

Kaplan–Meier analysis and Cox regression analysis were used to evaluate the prognostic value of ANXA2 expression in OSCC patients, as shown in Fig. 2 and Table 2. It was found that the median survival of OSCC patients in ANXA2 positive group was significantly shorter than that in ANXA2 negative group, indicating that ANXA2 positive expression may be associated with poor survival (*P* < 0.001). Moreover, TNM stage, lymph node metastasis and invasion depth were also correlated with the overall survival of OSCC patients (*P* < 0.001). Multivariate Cox regression analysis was conducted to analyze the further correlation of ANXA2 and OSCC, and the results showed that ANXA2 positive expression was an independent risk markers for poor prognosis of OSCC (Table 3).

**Table 2:** Kaplan–Meier and univariate analysis of all parameters in patients with OSCC.

Variables	Median survival (Month)	Log-Rank test		Cox univariable analysis	
		*χ*^*2*^	*P*	*HR* ( 95%*CI* )	*P*
**Age**					
≤ 57	47	1.215	0.270	1	
> 57	43			1.305(0.806 ~ 2.114)	0.280
**Gender**					
Male	47	0.102	0.749	1	
Female	44			1.081(0.666 ~ 1.752)	0.753
**Tumor size**					
< 3.3	47	1.420	0.233	1	
≥ 3.3	43			1.335(0.822 ~ 2.167)	0.246
**TNM stage**					
I + II	49	8.156	0.004	1	
III + IV	39			2.015(1.226 ~ 3.311)	0.006
**Degree of differentiation**					
Low	40	5.686	0.058	1	0.069
Moderate	37			1.402 (0.760 ~ 2.588)	0.279
High	52			0.711 (0.381 ~ 1.324)	0.282
**Lymph node metastasis**					
No	47	9.998	0.002	1	
Yes	37			2.310(1.345 ~ 3.967)	0.002
**Invasion depth**					
< 5 mm	46	4.183	0.041	1	
≥ 5 mm	40			1.811(1.008 ~ 3.254)	0.047
**Tumor location**					
Tongue	43	0.104	0.950	1	0.951
Gingiva	46			1.074 (0.613 ~ 1.881)	0.804
Others	48			1.103 (0.575 ~ 2.116)	0.768
**ANXA2 expression**					
Negative	54	13.676	0.000	1	
Positive	39			3.022(1.622 ~ 5.631)	0.000

**Table 3:** Cox multivariate analysis in patients with OSSC.

Variables	*B*	*SE*	*Wald*	*Sig*	*HR ( 95%CI )*
Lymph node metastasis	0.823	0.282	8.489	0.004	2.277 (1.309 ~ 3.960)
ANXA2 expression	1.110	0.323	11.812	0.001	3.034(1.611 ~ 5.712)

### Expression of ANXA2 in OSCC cells

**Figure 3:**
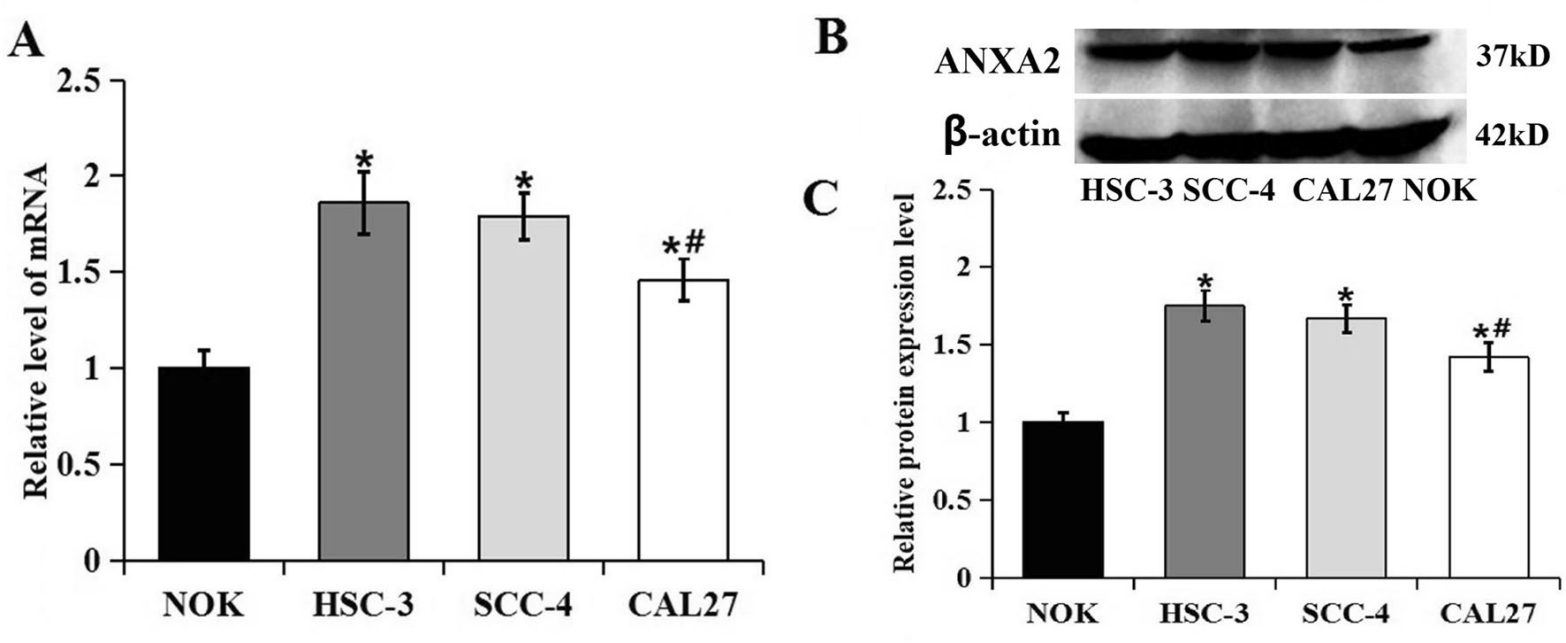
The mRNA and protein expression levels of ANXA2 in NOK, HSC-3, SCC-4 and CAL27. (**A**) RT-PCR showed mRNA expression of ANXA2 in the NOK, HSC-3, SCC-4 and CAL27 cells. (**B**) Western blot showed the protein expression of ANXA2 in the NOK, HSC-3, SCC-4 and CAL27 cells. (**C**) Comparison of the protein expression of ANXA2 in the NOK, HSC-3, SCC-4 and CAL27 cells. Data were presented as the mean ± SD of at least three repeated experiments. *P < 0.05, compared with NOK cells; ^#^*P* < 0.05, compared with HSC-3 and SCC-4 cells.

The results of PCR and western blot analysis showed that the expression of ANXA2 in OSCC cell lines HSC-3, SCC-4 and CAL27 were all higher than the normal NOK cells (Fig. 3), consistent with the results in tissues. Additionally, the expression of ANXA2 in HSC-3 and SCC-4 cells were higher than that in CAL27 cell, so HSC-3 and SCC-4 cells were selected for the further experiments.

### Silencing of ANXA2 suppressed the proliferation of OSCC cells

**Figure 4:**
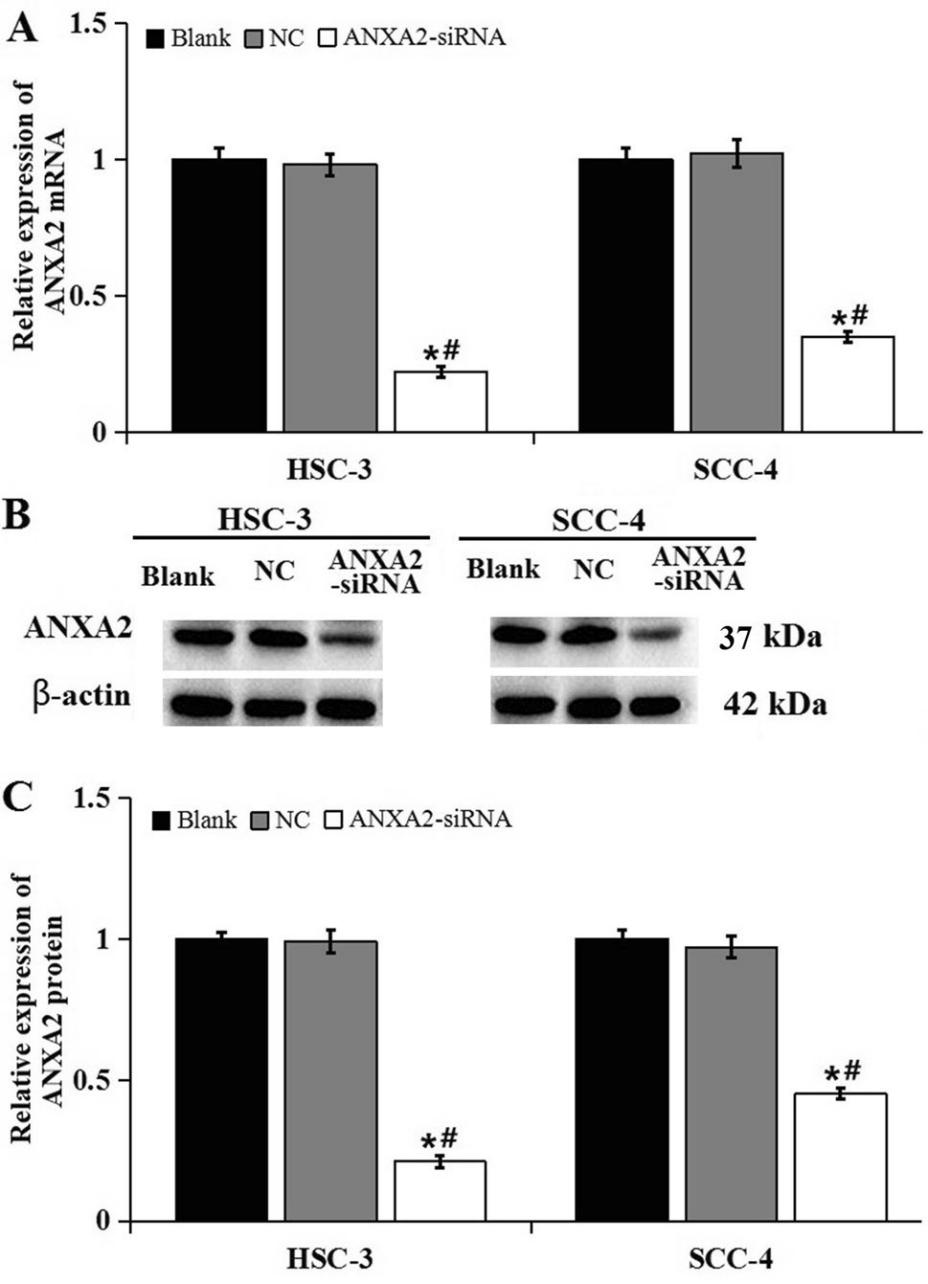
Expression of ANXA2 after transfection with siRNA. (**A**) RT-PCR showed mRNA expression of ANXA2 after transfection with siRNA in HSC-3 and SCC-4 cells. (**B**) Western blot showed the protein expression of ANXA2 after transfection with siRNA in HSC-3 and SCC-4 cells. (**C**) Relative expression of ANXA2 after transfection with siRNA in HSC-3 and SCC-4 cells. Data were presented as the mean ± SD of at least three repeated experiments. *P < 0.05, compared with blank group; ^#^*P* < 0.05, compared with NC group.

**Figure 5:**
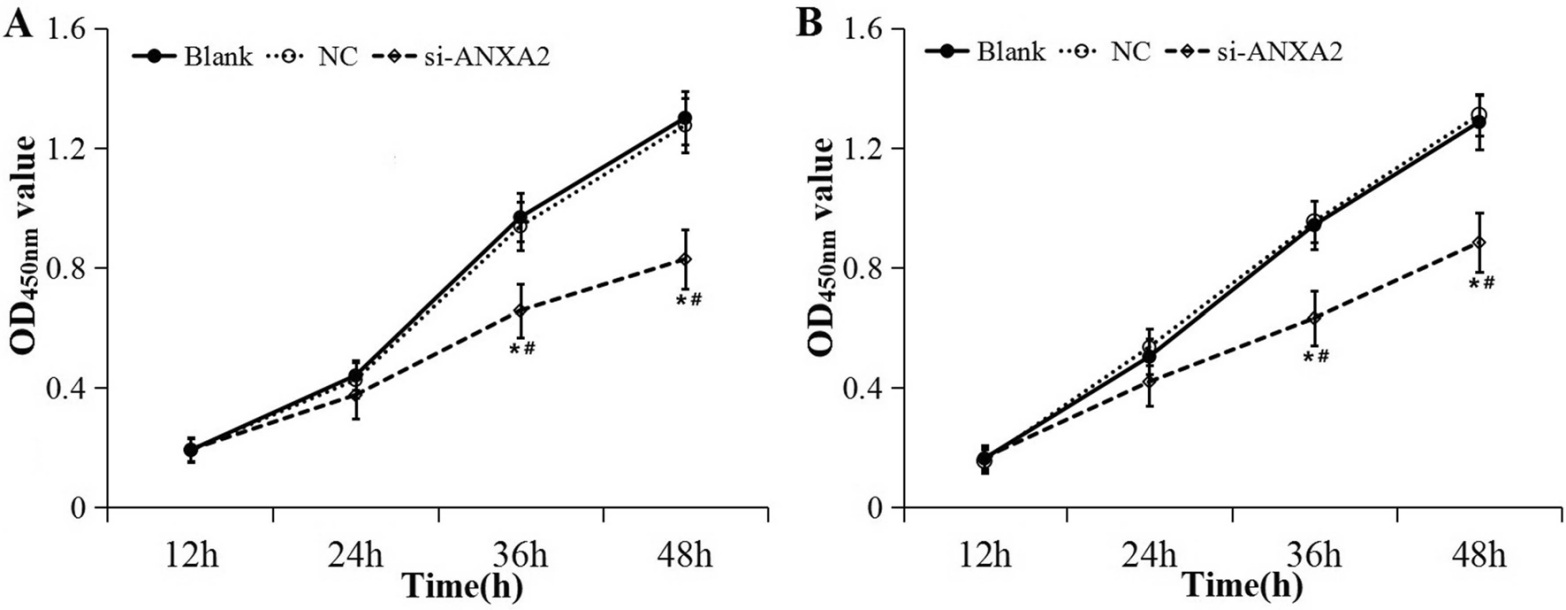
Effect of silencing ANXA2 expression on cell proliferation ability detected by CCK-8. (**A**) Cell proliferation ability of HSC-3 cells; (**B**) Cell proliferation ability of SCC-4 cells. Data were presented as the mean ± SD of at least three repeated experiments. *P < 0.05, compared with blank group; ^#^*P* < 0.05, compared with NC group.

As shown in Fig. 4, the expression level of ANXA2 mRNA and protein were significantly down-regulated in HSC-3 and SCC-4 cells after transfected with ANXA2-siRNA. Then CCK-8 assay was conducted to analyze the effect of ANXA2 on the proliferation ability of HSC-3 and SCC-4 cells (Fig. 5). The results showed that the proliferation activity in ANXA2-siRNA group was significantly lower than that in the NC and blank groups at 36 h and 48 h both in HSC-3 and SCC-4 cells (*P* < 0.05).

### Silencing of ANXA2 suppressed the migration ability of OSCC cells

**Figure 6:**
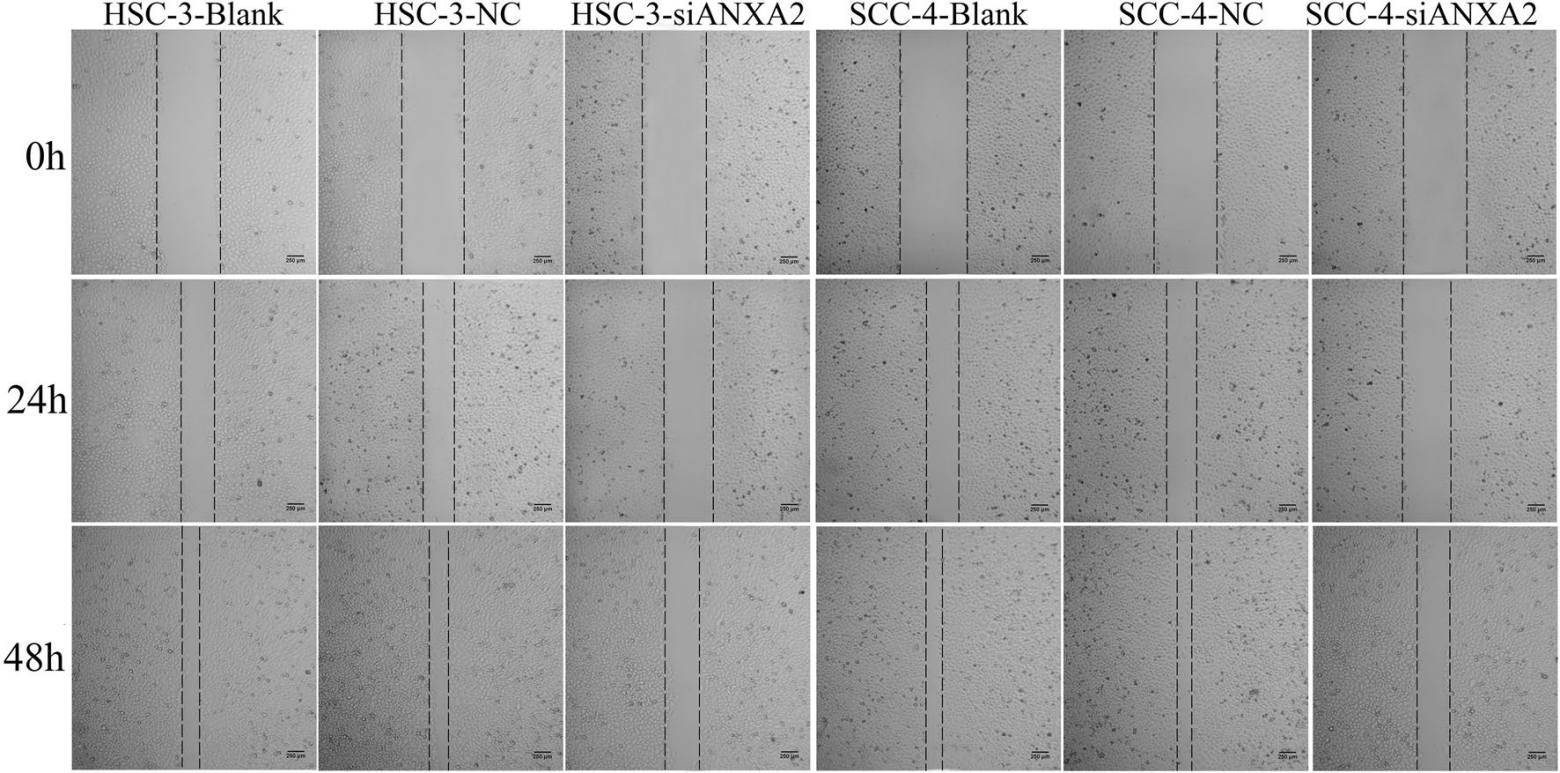
Effect of silencing ANXA2 expression on migration ability of HSC-3 and SCC-4 cells detected by wound healing assay.

Wound healing assay was used to identify the effect of ANAX2-siRNA on migration ability of HSC-3 and SCC-4 cells. As shown in Fig. 6, the migration rate of HSC-3 cells in the blank, NC and ANXA2-siRNA groups were (52 ± 4)%, (51 ± 5)%, and (32 ± 5)% at 24 h, (88 ± 4)%, (86 ± 5)%, and (62 ± 4)% at 48 h, respectively. The migration rate of SCC-4 cells in the blank, NC and ANXA2-siRNA groups were (50 ± 8)% , (48 ± 6)%, and (35 ± 5)% at 24 h, (90 ± 5)%, (89 ± 4)%, and (63 ± 5)% at 48 h, respectively. The results showed that ANXA2-siRNA significantly suppressed the migration of HSC-3 and SCC-4 cells (*P* < 0.05).

### Silencing of ANXA2 suppressed the invasion ability of OSCC cells

**Figure 7:**
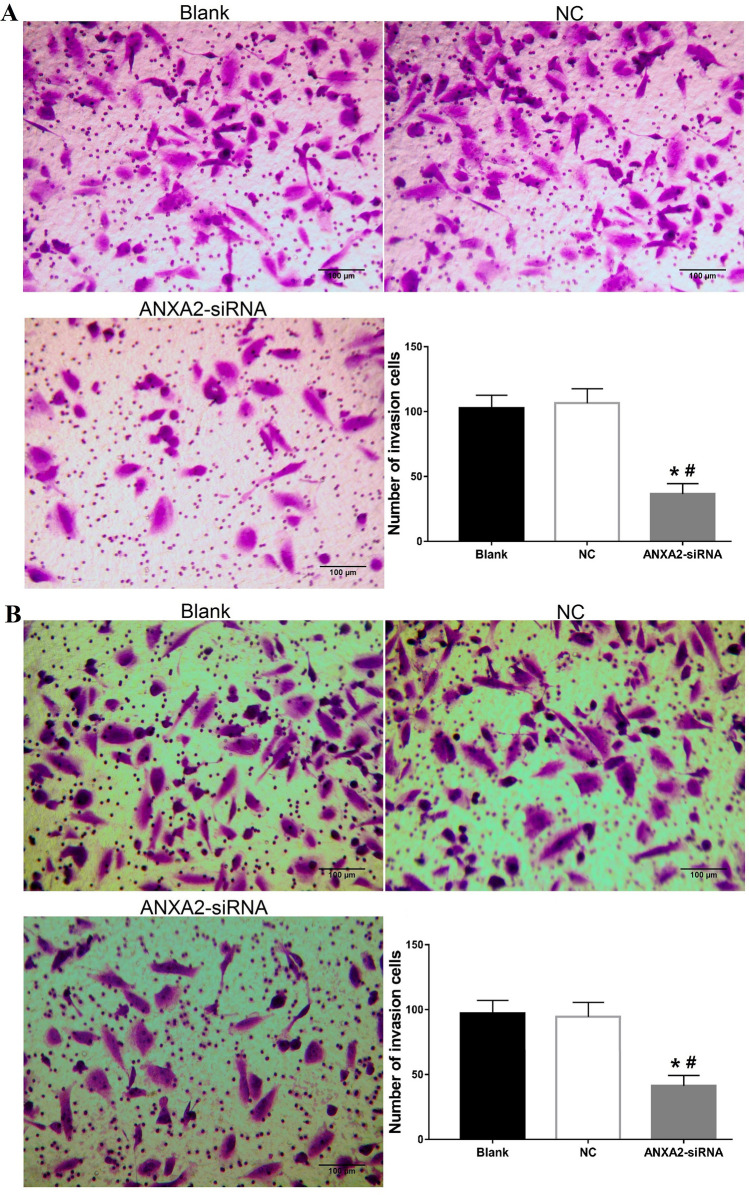
Effect of silencing ANXA2 expression on invasion ability of HSC-3 and SCC-4 cells measured by Transwell assay. (**A**) The invasion ability of HSC-3 cells in a visual field. (**B**) The invasion ability of SCC-4 cells in a visual field. Data were presented as the mean ± SD of five visual fields at least three repeated experiments. *P < 0.05, compared with blank group; ^#^*P* < 0.05, compared with NC group.

The invasion abilities of HSC-3 and SCC-4 cells were analyzed by Transwell assay. As shown in Fig. 7, the invasion cell numbers were significantly lower in the ANXA2-siRNA group compared with the NC and blank groups, indicating that ANXA2-siRNA suppressed the invasion ability of HSC-3 and SCC-4 cells.

## Discussion

OSCC is a malignant tumor with poor prognosis, and there is no ideal curative method currently. Understanding of the molecular mechanism of proliferation, invasion and metastasis of OSCC cells could provide useful effective therapeutic targets or prognostic indicators for this disease. ANXA2 has been reported to be associated with a variety of malignancies: A meta-analysis showed that ANXA2 over-expression was associated with tumor invasion, lymph node metastasis, overall survival, and poor prognosis^14^. Zhong et al.^15^ discovered that the expression of ANXA2 protein in OSCC tumor tissues was higher than that in adjacent non-malignant epithelial tissues which detected by immunohistochemistry. However, in Rodrigo’s research, the expression of ANXA2 in OSCC tissue was lower, compared with normal oral epithelial tissues^16^. In the present research, the expression of ANXA2 in OSCC tissues and adjacent carcinoma tissues were detected and verified that the positive expression rate of ANXA2 in OSCC tissues was higher than that in adjacent normal oral epithelium tissues, which was consistent with Zhong’s research^15^. Furthermore, the relationship of ANXA2 with OSCC patients’ clinical features were analyzed and the results indicated that the expression of ANXA2 was correlated with the TNM stage, tumor differentiation and lymph node metastasis (*P* < 0.05). In addition, Kaplan–Meier survival analysis found that patients with ANXA2 negative expression had a significantly longer overall survival than those with ANXA2 positive expression. And Cox multivariate analysis showed that ANXA2 positive expression was an independent risk factors for the survival of OSCC patients, which was in accordance with the results of Zhang et al.^17^. However, it still need to do more in-depth statistical analysis with large samples to verify the relationship of ANXA2 and prognosis and survival of OSCC patients.

In the process of tumor development, the changes of cytoskeleton, the decreased adhesion from cell to cell and cell to matrix increase the cell motility and invasion ability and promote tumor invasion and metastasis. In recent years, most studies have shown that ANXA2 played an important role in tumor cell growth, adhesion and metastasis^13^. In this research, the results showed that the expression of ANXA2 in OSCC cell lines HSC-3, SCC-4, CAL27 were higher than normal oral cell line NOK, which was consistent with the results in OSCC patients. The following experiments showed that knockdown of ANXA2 in OSCC cells inhibited the abilities of cell proliferation, migration, and invasion, suggesting that ANXA2 had positive effect on malignant biological properties of OSCC cell. The same results were observed in other cancers: The down-regulation of ANXA2 in lung cancer cell line A549^18^ or breast cancer cell line MDA-MB-231^19^ significantly reduced the proliferation capacity of tumor cells. Studies also found that silencing of ANXA2 inhibited cell proliferation by arresting cell cycle^20^. Wang et al.^18^ verified that the deletion of ANXA2 inhibited cell proliferation during cell cycle arrest by inducing p53. Ma et al.^12,21^ suggested that ANXA2 may regulate the invasion ability of cells through c-myc. And the further research demonstrated that ANXA2 activated the FIF1A-VEGF axis through increasing the protein abundance of myc, to promote cell migration and invasion^22,23^. Yuan et al.^24^ demonstrated that ANXA2 was correlated with the proliferation and invasion of breast cancer cells, and the mechanism was phosphorylation of ANXA2 enhanced STAT3 activation, increasing the expression of cyclin D1 and MMP2/9, which were the key target genes of STAT3 playing vital roles in cell proliferation and invasion. And Wu et al.^25^ indicated that ANXA2 increased the expression of c-myc and cyclin D1 by activating the Erk1/2 signaling pathway, thereby affecting the proliferation, migration, and invasion of MCF-7 cells. In mouse adenocarcinoma models, knockdown of ANXA2 significantly inhibited cell metastasis^26^. Andey et al.^27^ also found that silencing of ANXA2 gene could significantly reduce the metastasis ability of lung cancer cells. From the above studies, it can be concluded that ANXA2 might trigger different signaling pathways, promoting the proliferation, invasion and metastasis ability of tumor cells, and accelerating the development of tumor. The results in the present study show that ANXA2 is significant for the development and survival of OSCC and plays a pivotal role in tumor proliferation, migration, and invasion of OSCC cells, which should be concerned by clinical and considered as a potential target of OSCC therapy. However, specific mechanism and possible involved signal pathways have not been studied. Therefore, it is necessary to further explore the mechanism of ANXA2 in OSCC.

## Conclusions

The present study showed that ANXA2 positively expressed in most OSCC tissues, and was correlated with the TNM stage, tumor differentiation and lymph node metastasis in OSCC. Knockdown of ANXA2 expression inhibited the proliferation, migration and invasion of OSCC cells. These results indicated that ANXA2 may play a pivotal role in OSCC progression and may be a potential target for therapeutic intervention of OSCC.

## Material and methods

### Patients and samples

OSCC and adjacent specimens were collected from 124 OSCC patients who visited Henan Provincial Stomatological Hospital from Oct, 2014 to Oct, 2017. All patients were primary OSCC with complete medical records, and the pathological specimens were obtained by resection operation and well preserved in liquid nitrogen. None of the patients had a history of chemoradiotherapy or immunotherapy, without any autoimmune diseases or oral mucosal diseases. Patients were followed up 24–60 months, until death or Oct, 2019. Overall survival time referred to the beginning of treatment to death or the last follow-up. 124 patients included 56 males and 68 females with an age of (55.5 ± 1.65) years old (range: 30 ~ 76 years). All patients had signed the informed consent for participating in the research and the research has been approved by the Ethics committee of the First Affiliated Hospital of Zhengzhou University.

### Cell culture

Normal Oral corneum cell line NOK and Human oral squamous cell carcinoma cell lines HSC-3, SCC-4 and CAL27 were given by School of Stomatology, Zhengzhou University. NOK, HSC-3 and CAL27 cells were cultured in MEM (41,500,034, Gibco, USA), and SCC-4 cells were cultured in DMEM/ Nutrient Mixture F-12 (DMEM/F12, 1:1) medium (A4192001, Gibco, USA), all of them containing 10% FBS (04-001-1A-001AUS, Bioind, Israeli), penicillin (100 U/L) and streptomycin (100 mg/L) (SV30010, HyClone, USA). The cells were cultured in an incubator of 5% CO_2_ at 37 °C. The medium was replaced once in 2 days.

### Immunohistochemistry (IHC) for detecting ANXA2 expression in tissues

Tissues removed during surgery were fixed with 10% neutral formalin solution, dehydrated and embedded in paraffin and cut into 4 μm sections. Then the sections were deparaffinized, rehydrated, and subjected to microwave antigen retrieval in citrate buffer for 20 min. Then treated with 3% H_2_O_2_ for 30 min to block endogenous peroxidase. To reduce non-specific binding, FBS treatment was used for 30 min. Then a rabbit polyclonal ANXA2 antibody (Y055296, abm, Canada) was incubated for 2 h at 36 °C. After washed with PBS, the sections were incubated with a goat anti rabbit IgG antibody (SE134, Solaribio, Beijing, China) for 30 min. At last, the sections were washed with PBS and color reacted with DAB (Solarbio, Beijing, China) as a chromogen. The sections were counterstained by hematoxylin solution, dehydrated. And the sections were photographed under light microscope (OLYMPUS, CX31, Japan). The expression of ANXA2 was defined according to the staining intensity, which was scored as ‘0’ (0 ~ 25%), ‘1’ (26% ~ 50%), ‘2’ (51% ~ 75%), ‘3’ (76% ~ 100%). 5 fields of view were selected to measure the staining intensity for each section, and the average score from 3 sections was represented as the score of ANXA2 expression. The final score of 0 ~ 1 defined as negative and 2 ~ 3 was defined as positive.

### qRT-PCR analysis for detecting ANXA2 mRNA expression level in cells

RNA was extracted according to the Trizol-spin-column kit (Generay, Shanghai, China) protocols and reversed transcribed using the HiScriptII QRT SuperMix for qPCR kit (Vazyme, Nanjing, China). The expression level of ANAX2 was determined by using ChamQ SYBR Color qPCR Master Mix kit (Vazyme, Nanjing, China). PCR amplification was performed on a CFX Connect Real-Time System (BIO-RAD, USA). Primer sequences were as follows: ANXA2-F: AAATGTCTACTGTTCACGAAATC; ANXA2-R: GTGTCGGGCTTCAGTCATC; β-actin-F: ACTCTTCCAGCCTTCCTTCC; β-actin-R: CATACTCCTGCTTGCTGATCC. 6 multiple holes were set in each experiments and the experiment was repeated three times. The two of three cancer cell lines whose ANXA2 expression level were higher were selected to do the following experiments.

### Western blot for detecting ANXA2 protein expression in cells

The cells about 1 × 10^6^ were collected at 4 °C, 1200 rpm centrifugetion, and washed by ice-cold PBS twice. And 500 µl RIPA buffer (containing PMSF) was added into the cells and centrifuged at 4 °C, 13,000 rpm for 10 min to extract the total protein. The protein concentration was detected by BCA method. SDS loading buffer was added in protein specimen and degenerated at 100 °C for 5 min. 40 µg protein was separated by 12% SDS-PAGE, and transferred to PVDF membrane by wet type galvanometer (BIO-RAD, USA). The membrane was blocked by 5% skim milk for 1 h at room temperature, and cut according to precision plus protein dual color standards value (protein marker, BIO-RAD, USA) to incubate the antibodies. Then the primary antibodies (abm, Canada): Anti-ANXA2 (Y055296, 1:1000), β-actin (G046, 1:2000) were incubated at 4 °C overnight. The secondary antibody (ab6721, 1:5000, Abcam, USA) were incubated for 1 h. ECL detection reagent was added to the membranes to observe the protein bands, and the band densities were analyzed by Image J software (National Institutes of Health, Bethesda, USA,V 1.48, https://imagej.en.softonic.com/) with β-actin as internal reference. The experiment was repeated three times.

### Transfection of siRNA to silencing the ANXA2 expression

Human OSCC cell lines HSC-3 and SCC-4 were transfected with scrambled siRNA (Negative Control) and human ANXA2-specific siRNA (ANXA2-siRNA) respectively, which were designed and purchased from GenePharma (Shanghai, China). The transfection of siRNAs was performed using RFect siRNA transfection Reagent (BAIDAI, Changzhou, China) according to the manufacture’s protocol. After transfected for 24 h, qRT-PCR analysis was used to detect the expression of ANXA2 mRNA. And after transfected for 48 h, western blot analysis was used to detect the expression of ANXA2 protein to verify the transfection efficiency.

### CCK-8 assay for detecting cell proliferation

Cell proliferation was measured using a CCK-8 Cell Counting Kit (Vazyme, Nanjing, China). After transfected for 24 h, cells were seed into 96-well plates at 1 × 10^4^ cells/well with 3 replicate wells per group. After cultured for 12, 24, 36, and 48 h, 10 μl CCK-8 solution was added to each well and incubated for 2 h. The absorbance OD was measured at 450 nm by the microplate reader (BioTek, Winooski, USA). The experiment was repeated three times.

### Wound healing assay for detecting cell migration ability

After transfected for 24 h, cells were grown in 6-well plates at 1 × 10^6^ cells/well. When cells were at 80% confluency, a two-hundred-microliter-pipette was used to create a wound, and the cells were cultured in fresh serum-free medium. The healing of the scratch area was observed and photographed under an inverted microscope at 0, 24, and 48 h respectively. The healing area was calculated by Image J software.The migration ability was evaluated by healing area (%): (Initial scratch width—observation scratch width)/Initial scratch width × 100%. The experiment was repeated three times.

### Transwell assay for detecting cell invasion ability

The Transwell upper chamber (catalog 3422, Corning, USA) were coated by Matrigel (BD Biosciences, USA). The transfected cells were digested with 0.25% trypsin and collected at 1500 rpm centrifugation for 5 min. Cells were resuspended with MEM medium containing 2% FBS to adjust the cell density at 1 × 10^6^ cells/ml. And 200 μl cells were added into the upper chamber. The MEM medium with 10% FBS were added into lower chamber. After 24 h, the cells passed through the Matrigel-coated membrane (8.0 μm pore size) were fixed, stained with 0.1% crystal violet for 15 min. Then cells were counted using a microscope (OLYMPUS, Tokyo, Japan). Five visual fields were randomly selected to calculate the numbers of invaded cells.

### Statistical analysis

All the data were analyzed by SPSS 20.0. The measurement data was showed as mean ± standard deviation and the comparison between two groups was analyzed by independent sample *t-*test; For enumeration data, comparison between two groups was analyzed by chi-square test, *P* < 0.05 was considered statistically significant. Survival analysis was performed by Kaplan–Meier analysis, univariate and multivariate Cox regression analysis.

### Ethical statement

The present study was conducted with the informed consent of all participates, all experiments were performed in accordance with relevant guidelines and regulations of Declaration of Helsinki and has been approved by the ethics committee of the first affiliated hospital of Zhengzhou University.

## Supplementary Information

Supplementary Information

## Data Availability

The datasets generated during the current study are available from the supplementary files.
