# Supplementary material for: Clinical significance of Annexin A2 expression in oral squamous cell carcinoma and its influence on cell proliferation, migration and invasion
Source: Sci Rep. 2021 Mar 3;11:5033. doi: 10.1038/s41598-021-84675-y (PMC7930260; doi:10.1038/s41598-021-84675-y)
Supplement: Supplementary file 1 — Supplementary Information [file 41598_2021_84675_MOESM1_ESM.pdf]

Clinical significance of Annexin A2 expression in oral squamous cell carcinoma and its influence on cell proliferation, migration and invasion

Yingyi Ma[1], Haiye Wang[2]

[1]Department of Periodontal, The First Affiliated Hospital of Zhengzhou University, Henan Provincial Stomatology Hospital, Zhengzhou 450052, Henan Province, China.

[2]Special department, The First Affiliated Hospital of Zhengzhou University, Henan Provincial Stomatology Hospital, Zhengzhou 450052, Henan Province, China.

Corresponding to: Haiye Wang

E-mail: wh_zyfck@163.com

Address: Special department, The First Affiliated Hospital of Zhengzhou University, Henan Provincial Stomatology Hospital, No.1 Jianshe East Road, Zhengzhou 450052, Henan Province, China.

Tel: +8615838126816.

Transwell-HSC-3-Blank(1)

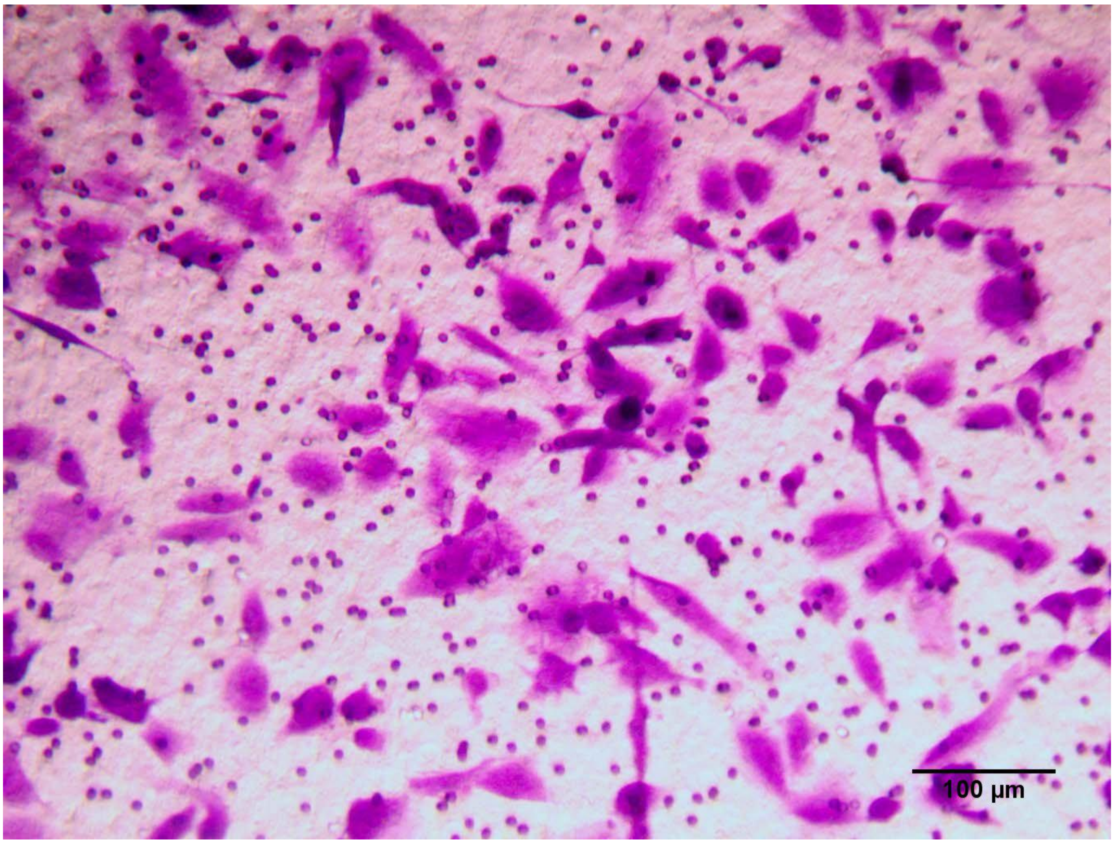

Transwell-HSC-3-Blank(2)

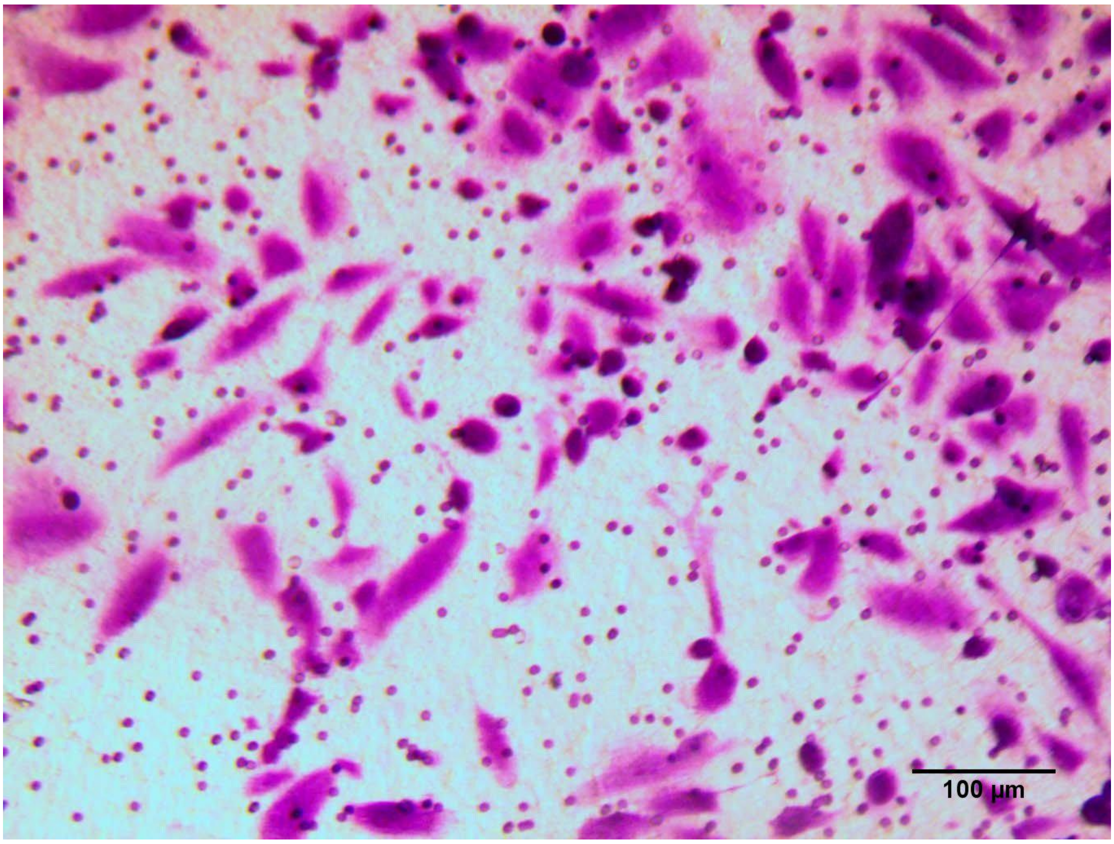

Transwell-HSC-3-Blank(3)

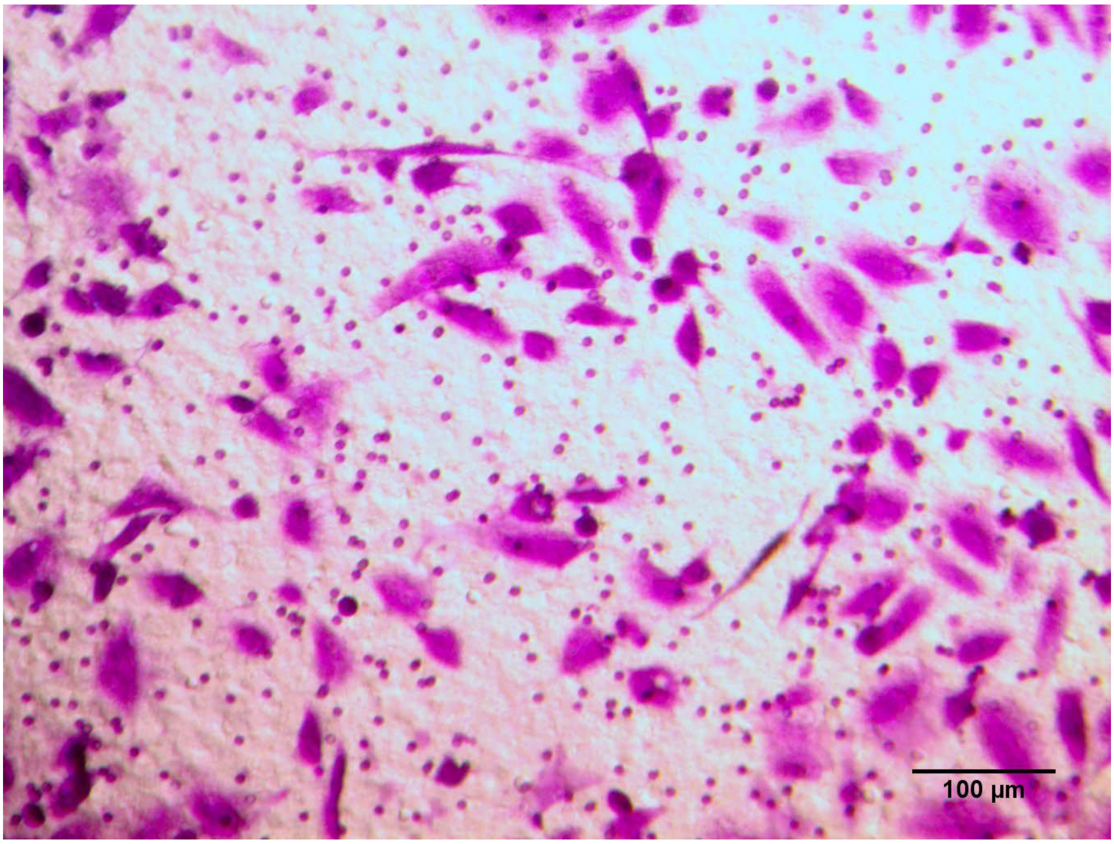

Transwell-HSC-3-Blank(4)

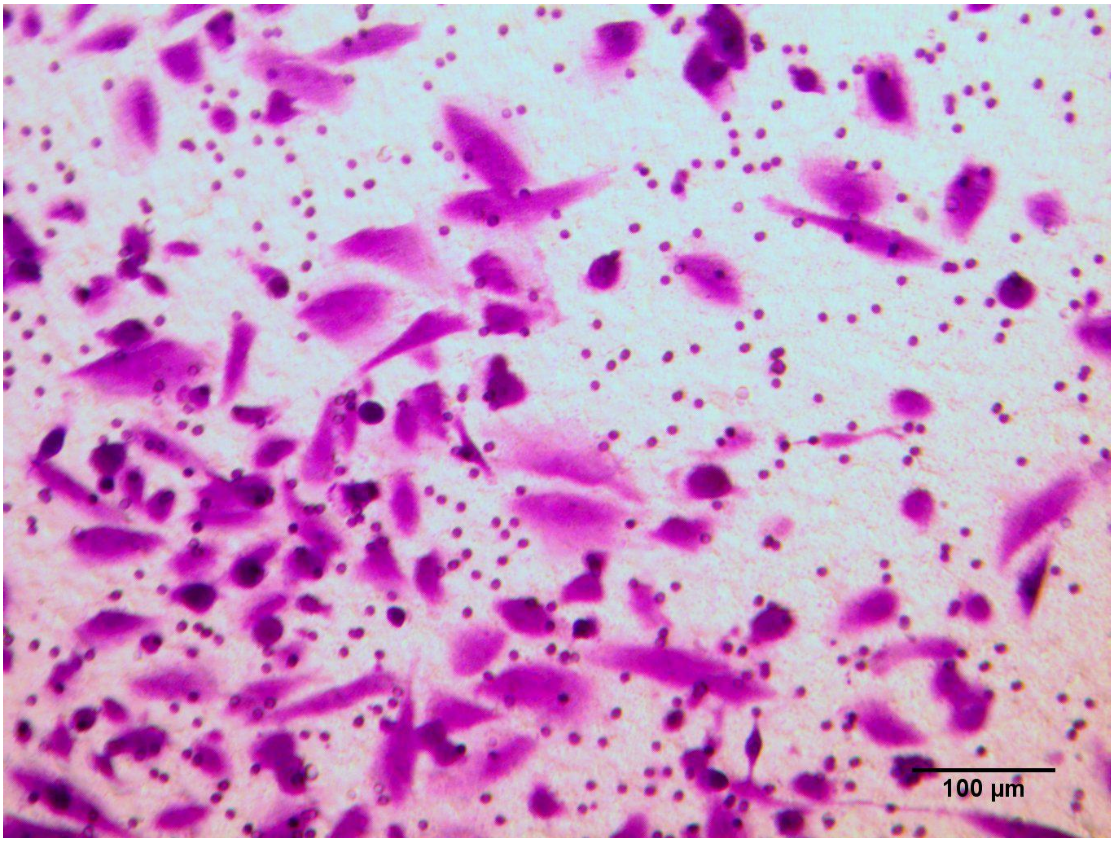

Transwell-HSC-3-Blank(5)

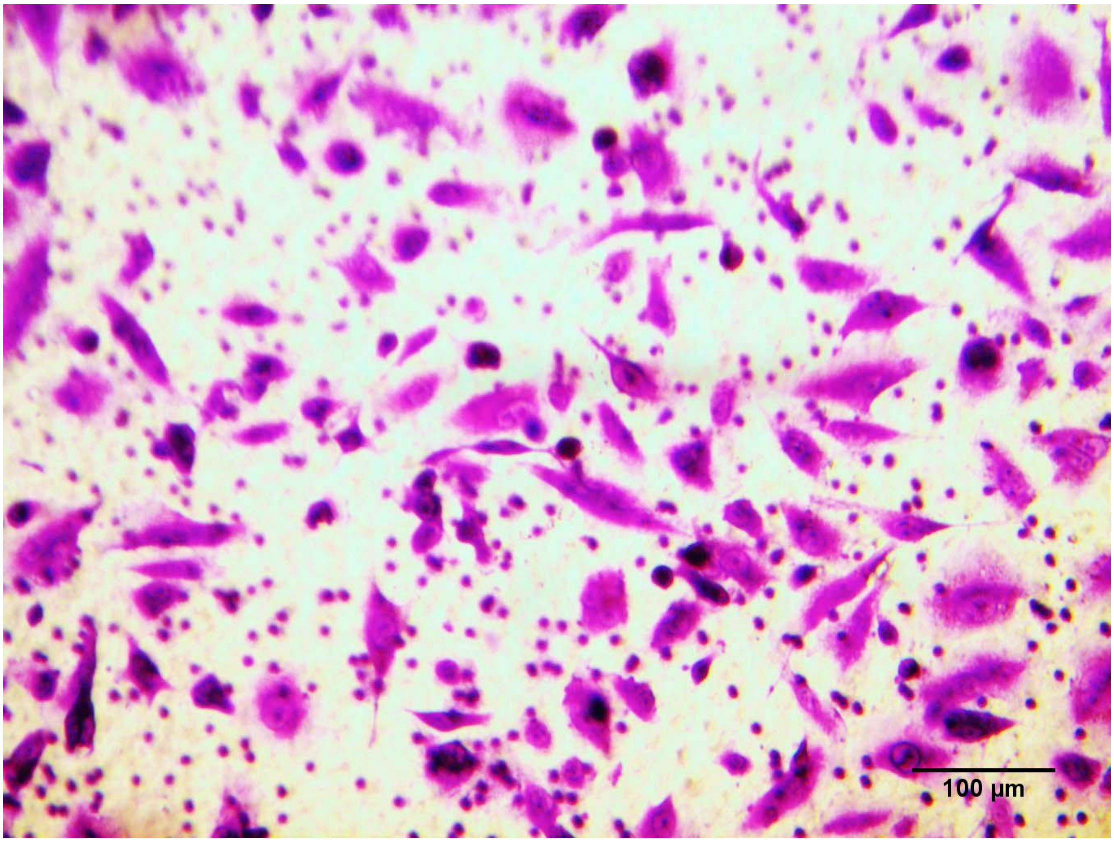

Transwell-HSC-3-NC(1)

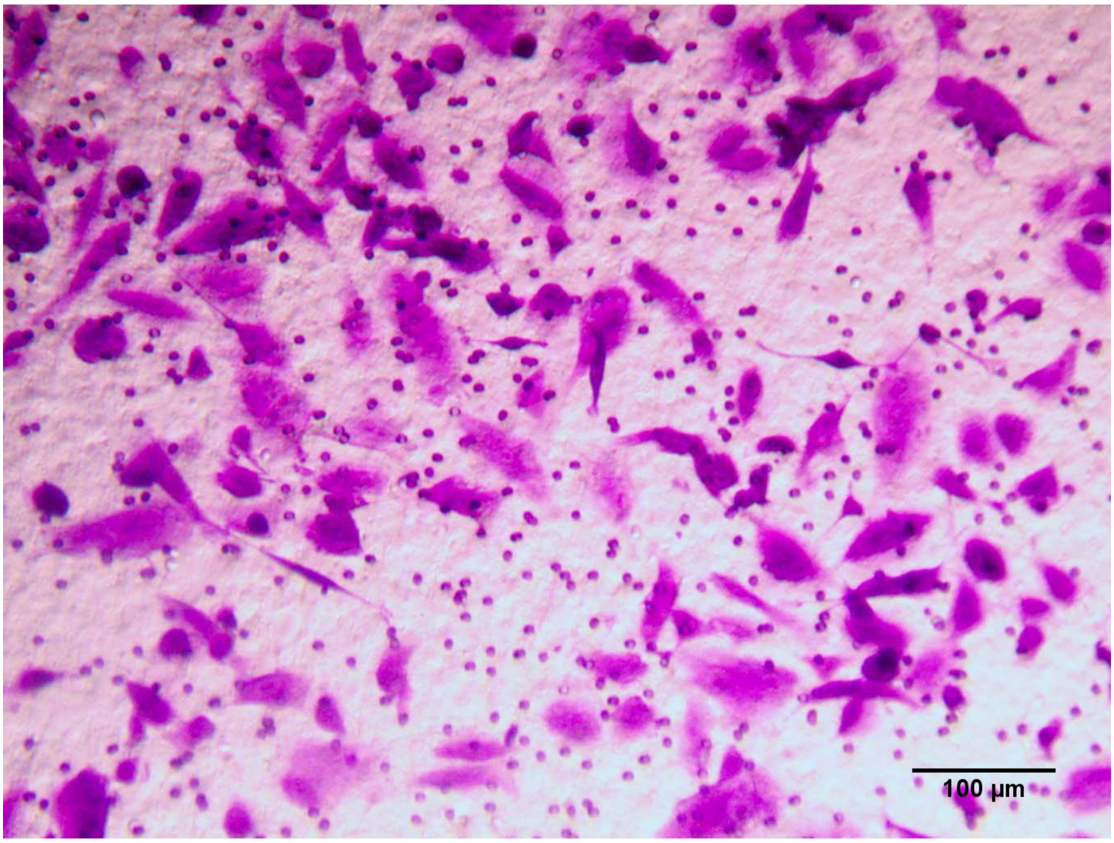

Transwell-HSC-3-NC(2)

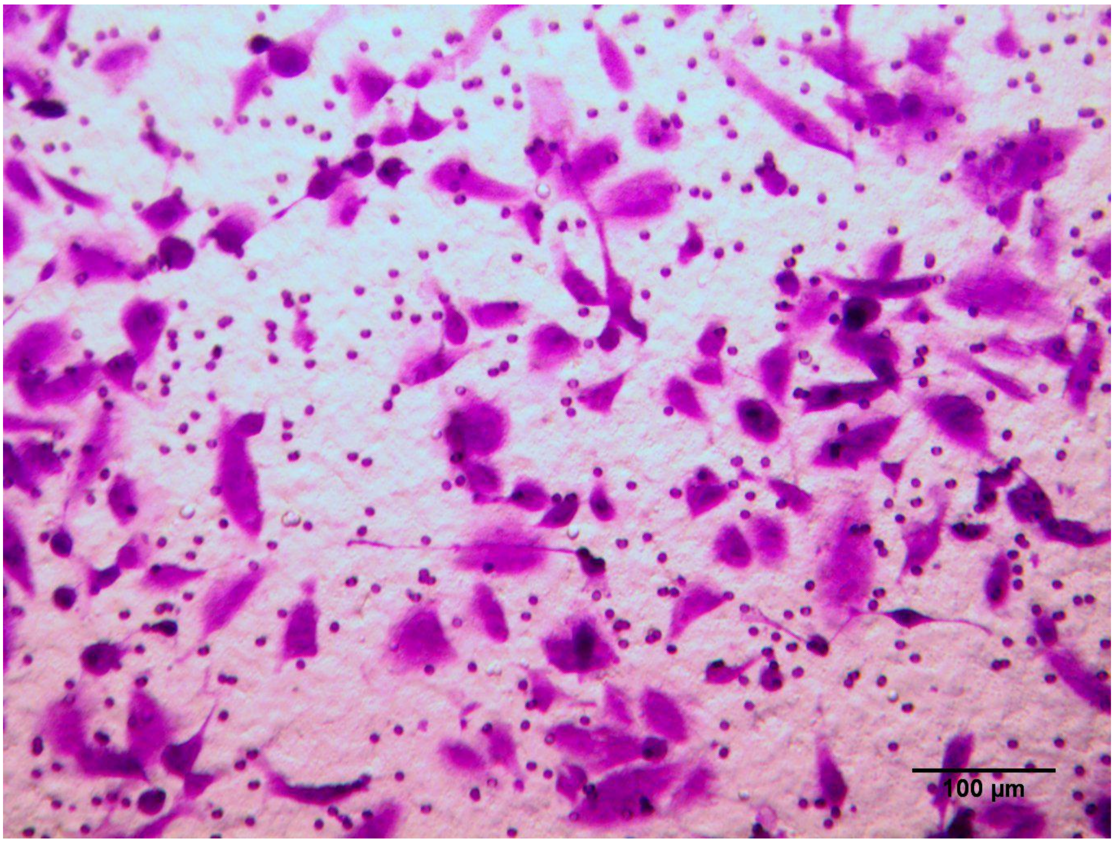

Transwell-HSC-3-NC(3)

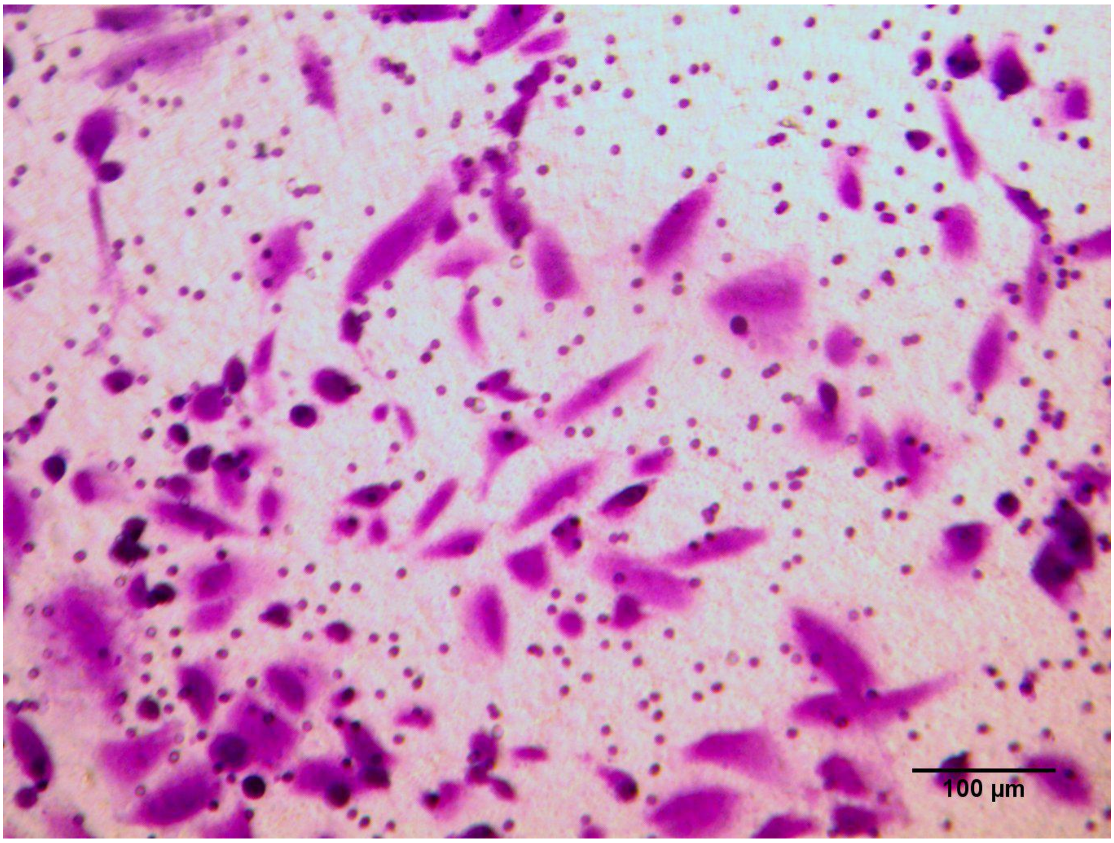

Transwell-HSC-3-NC(4)

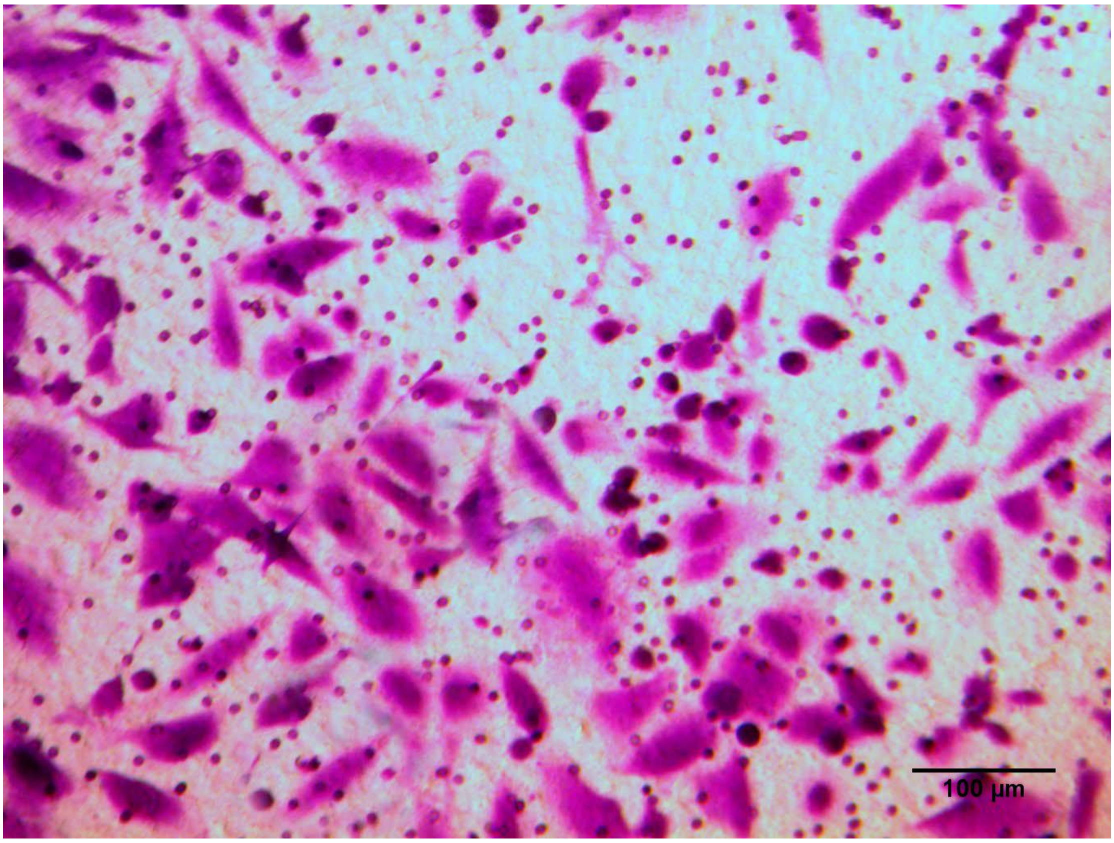

Transwell-HSC-3-NC(5)

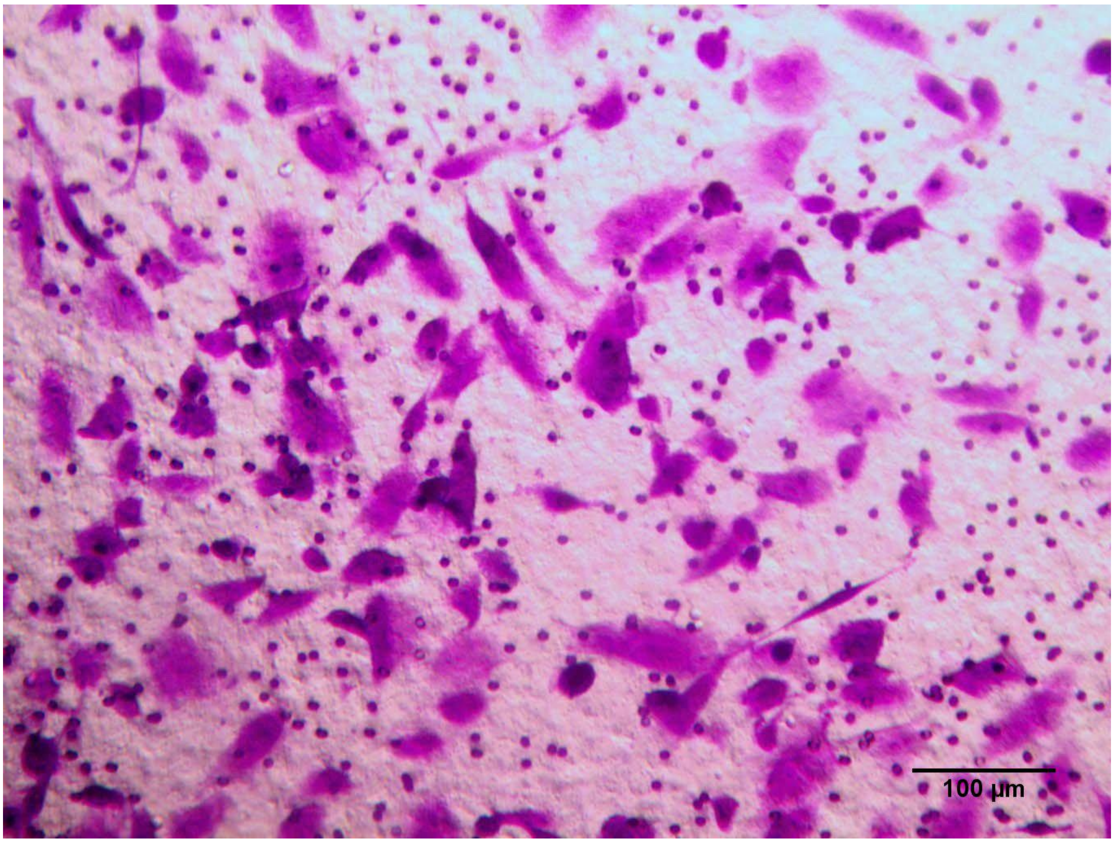

Transwell-HSC-3-siRNA(1)

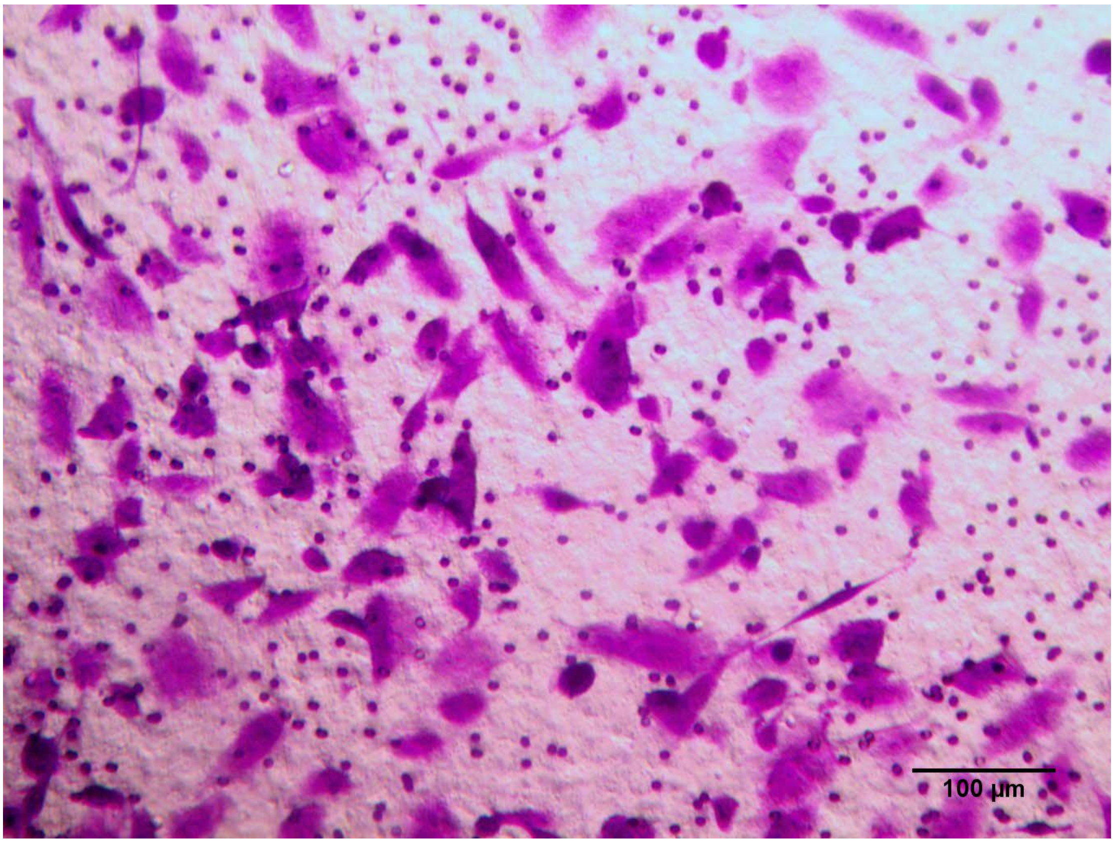

Transwell-HSC-3-siRNA(2)

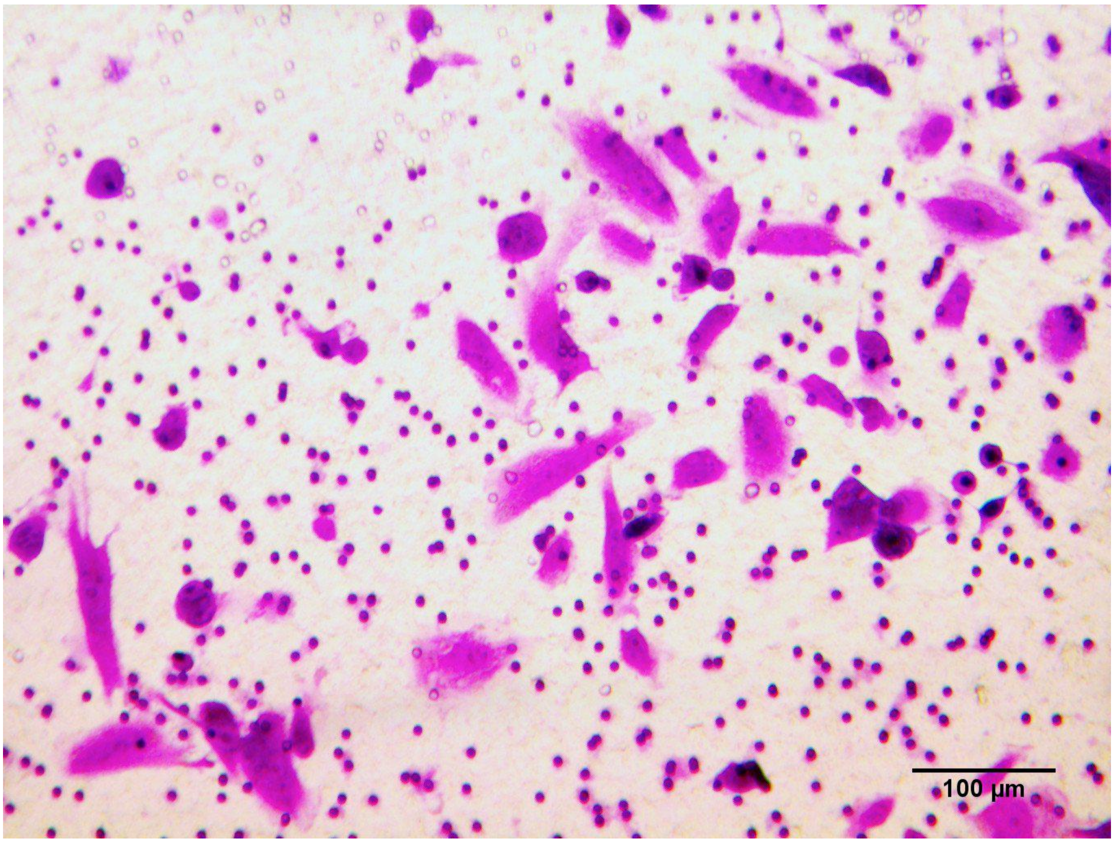

Transwell-HSC-3-siRNA(3)

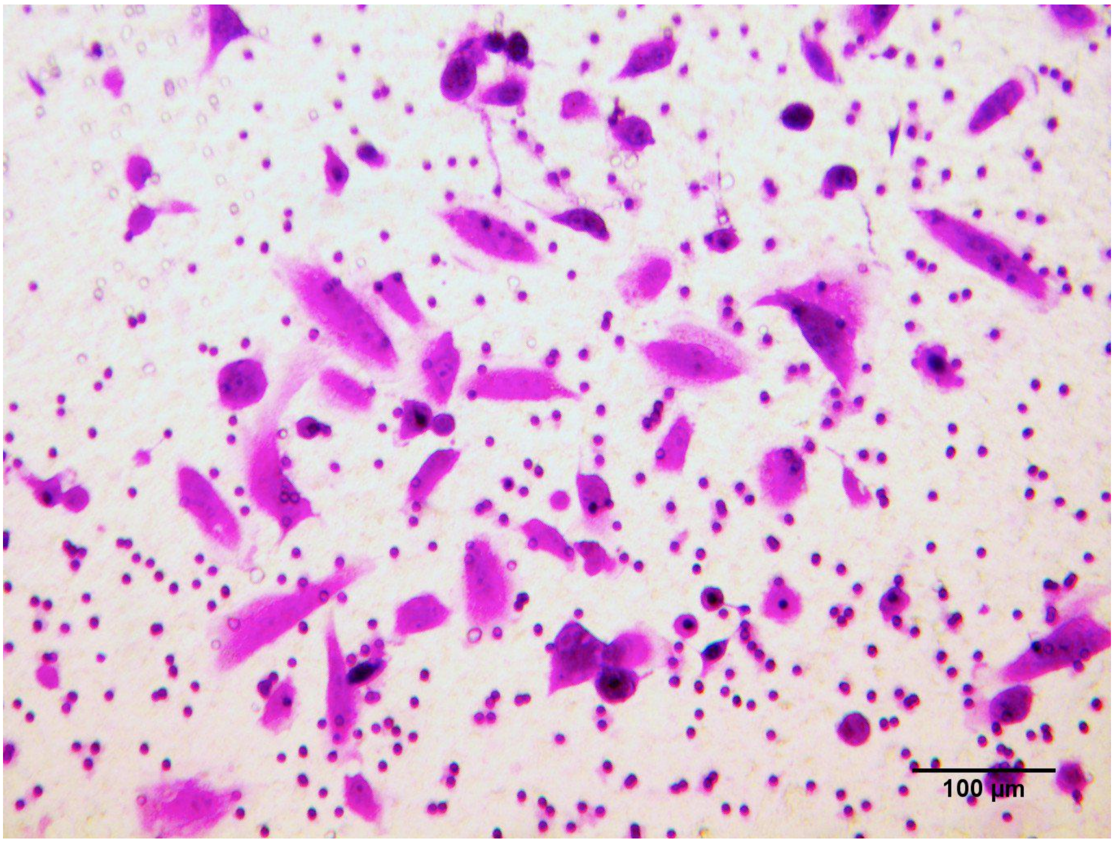

Transwell-HSC-3-siRNA(4)

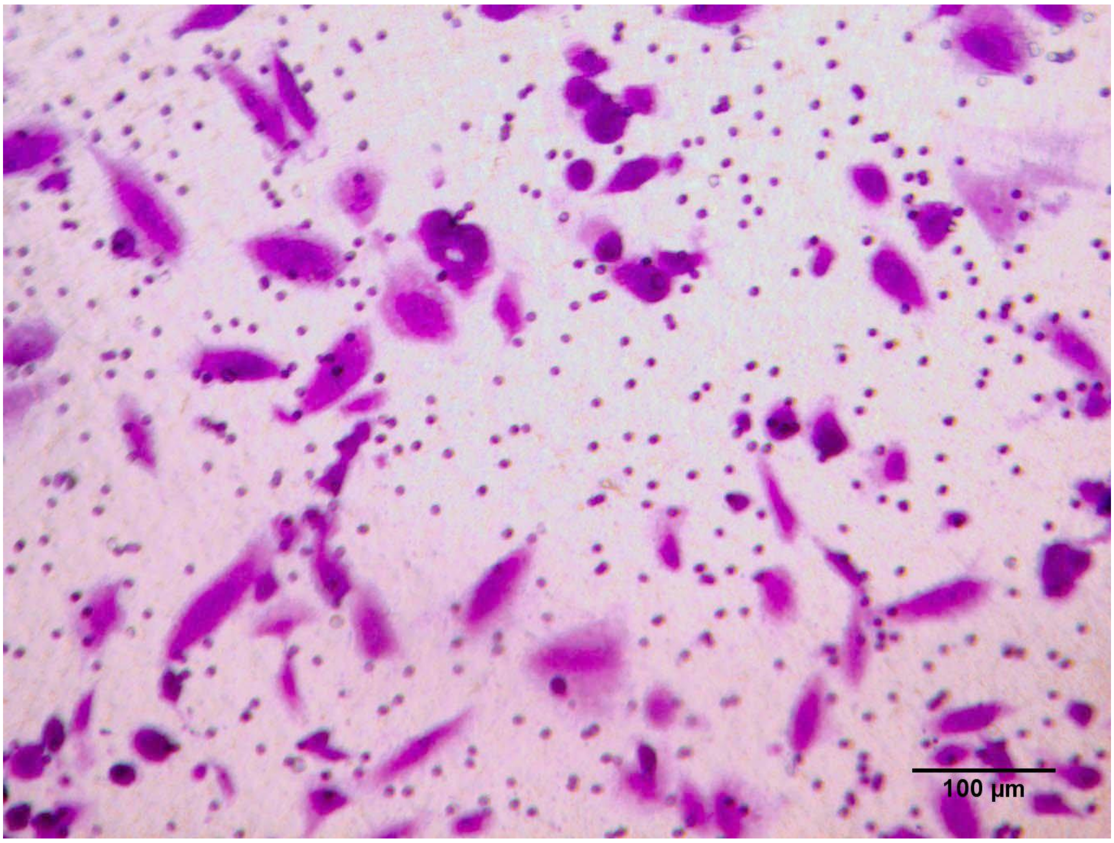

Transwell-HSC-3-siRNA(5)

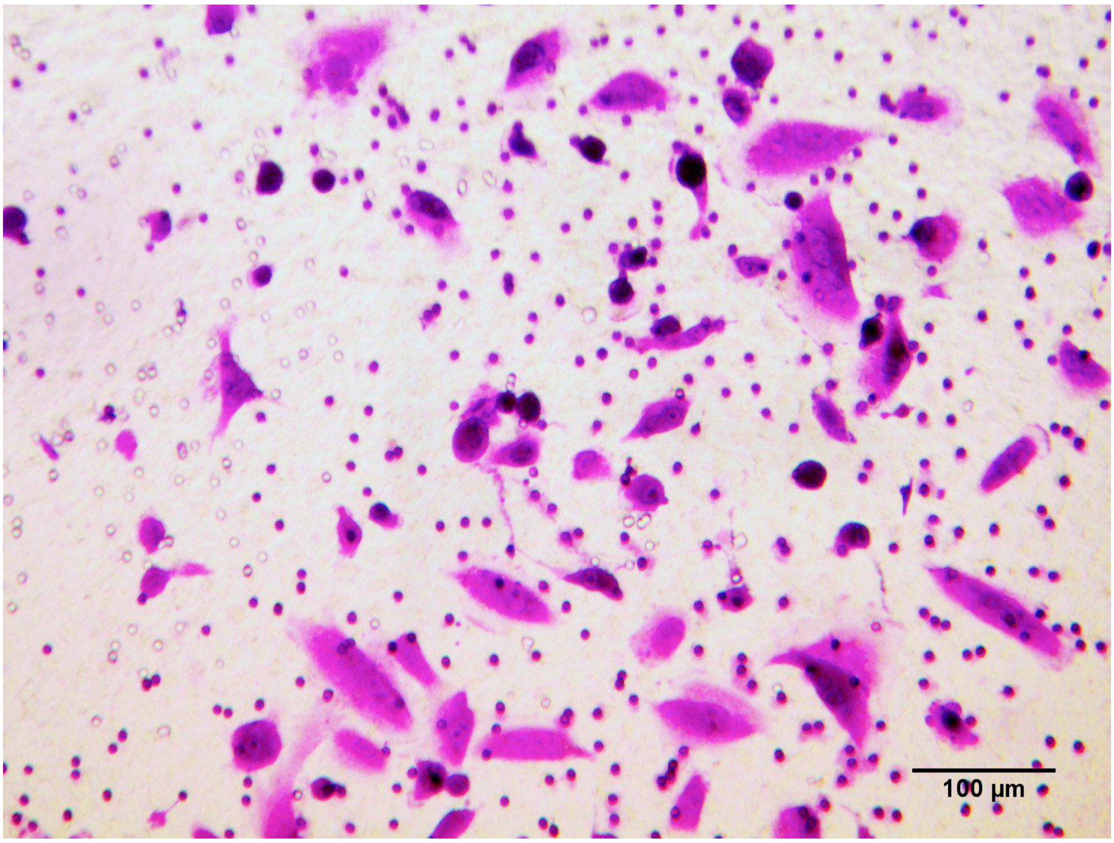

Transwell-SCC-4-Blank(1)

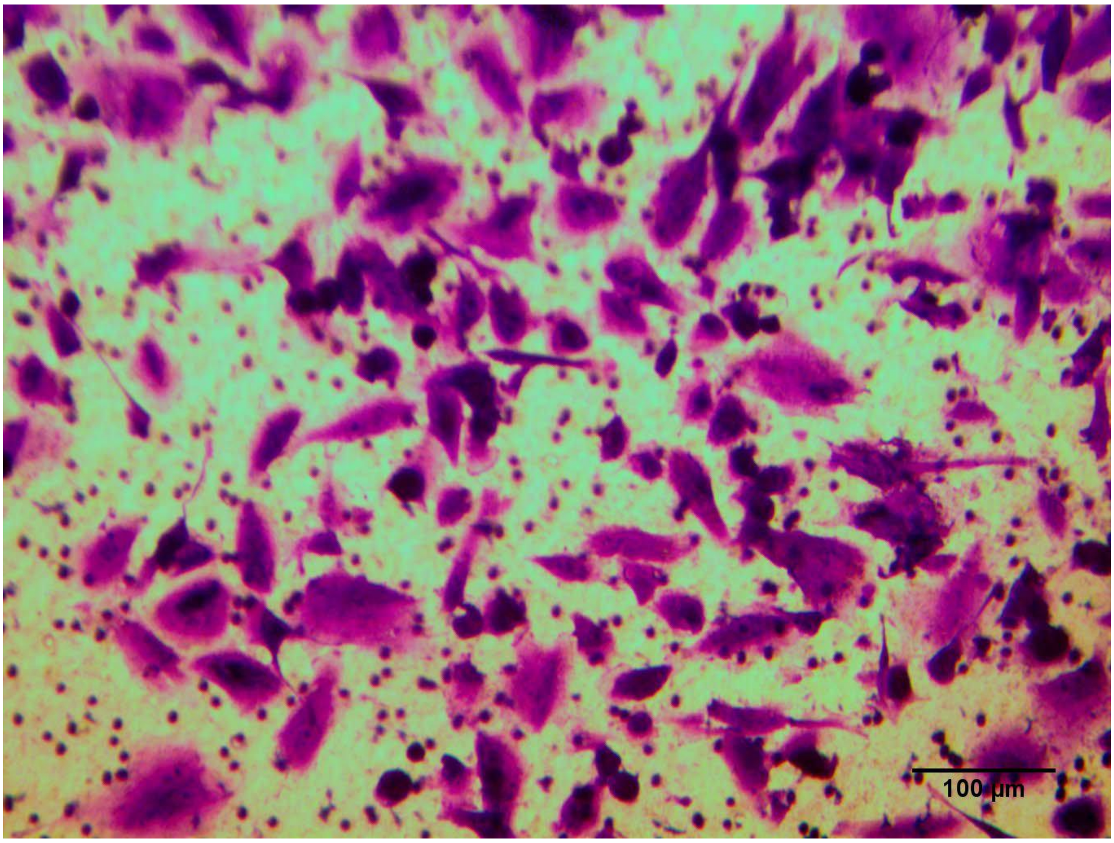

Transwell-SCC-4-Blank(2)

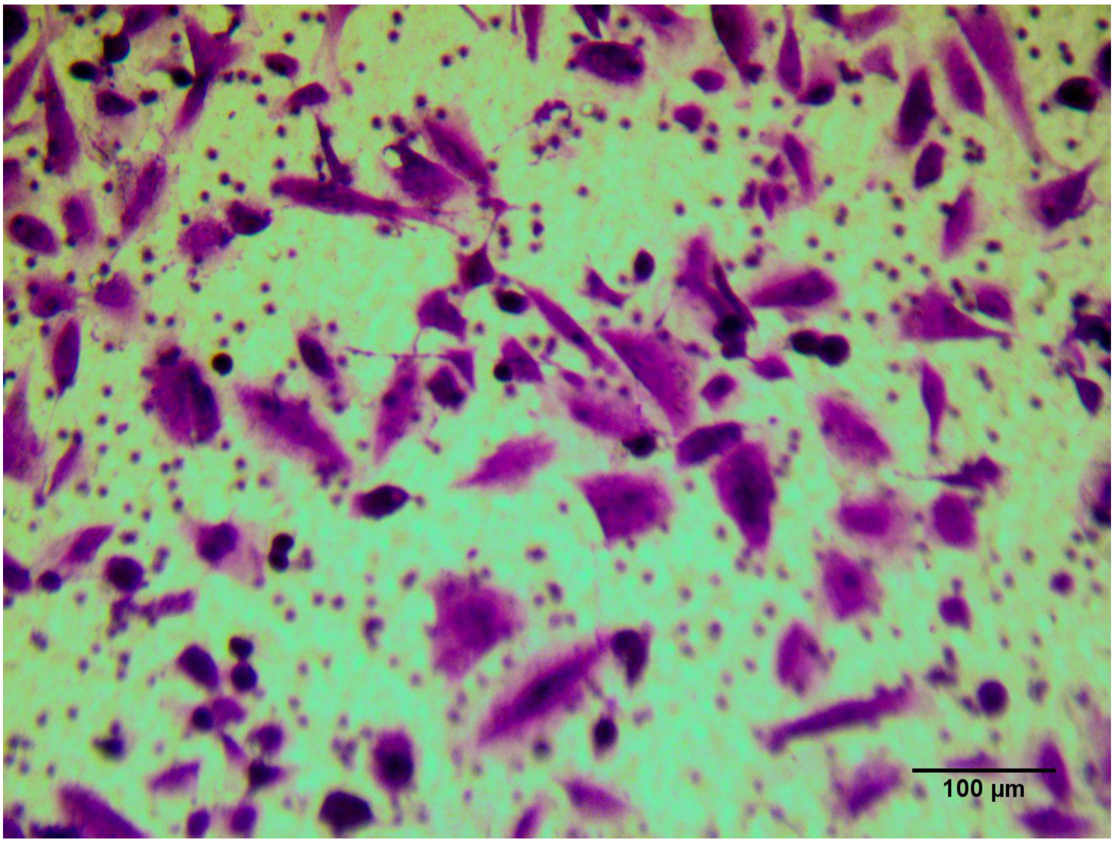

Transwell-SCC-4-Blank(3)

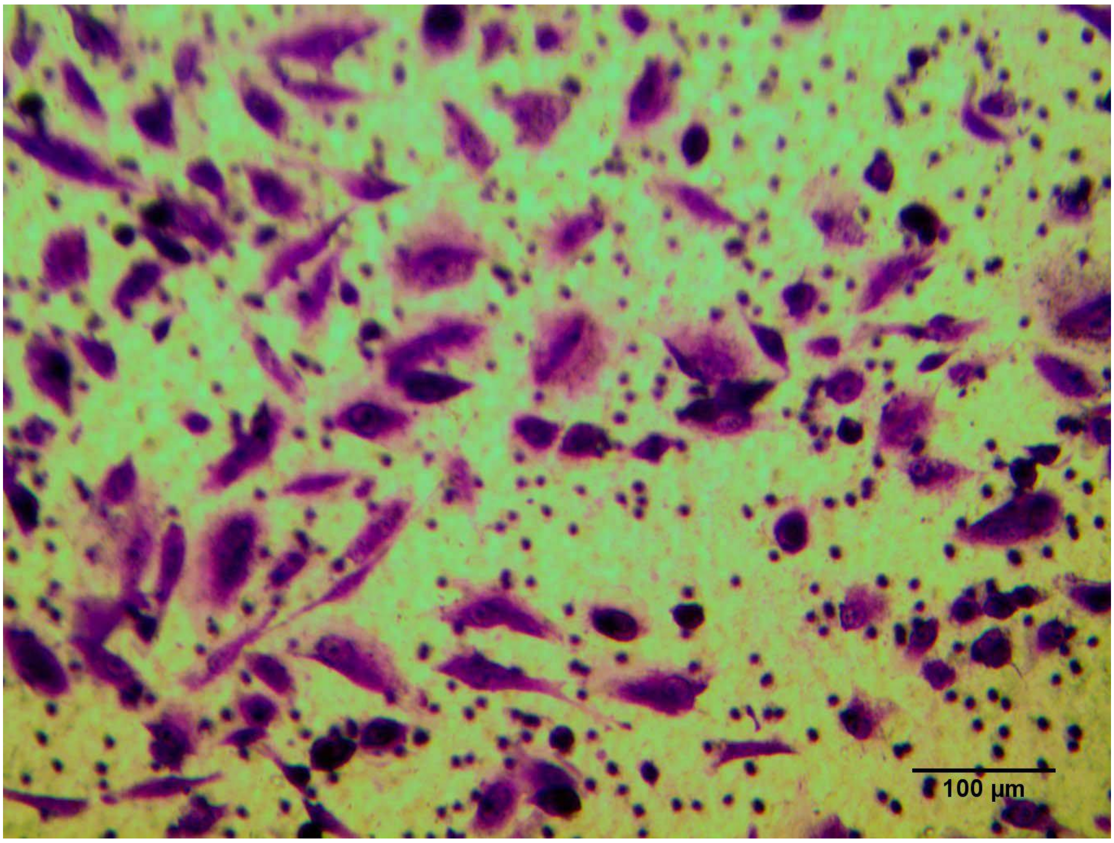

Transwell-SCC-4-Blank(4)

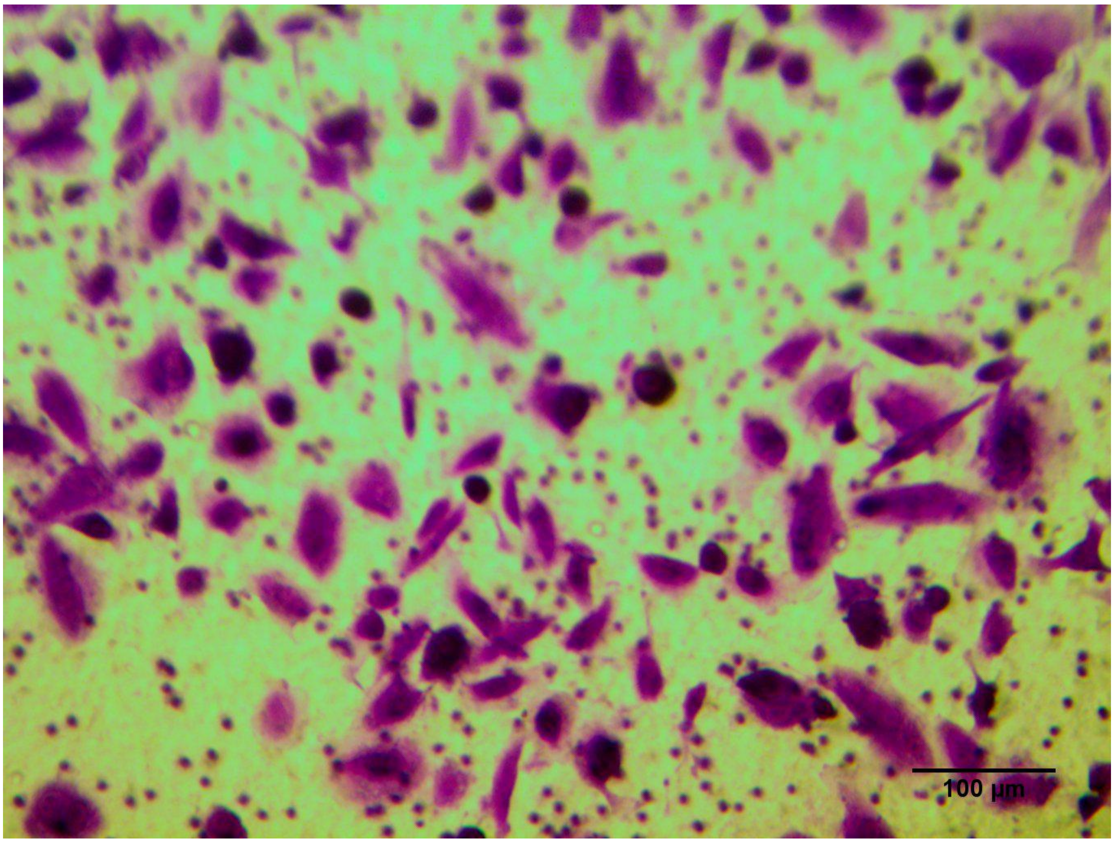

Transwell-SCC-4-Blank(5)

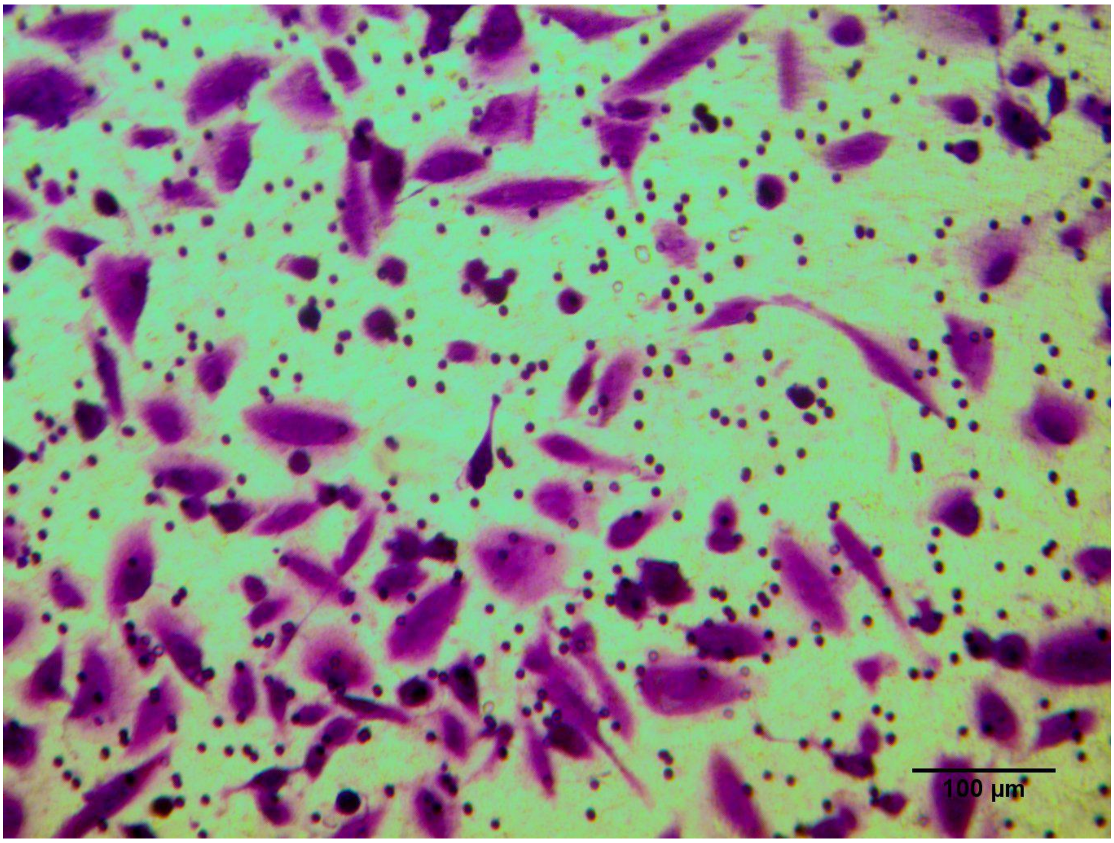

Transwell-SCC-4-NC(1)

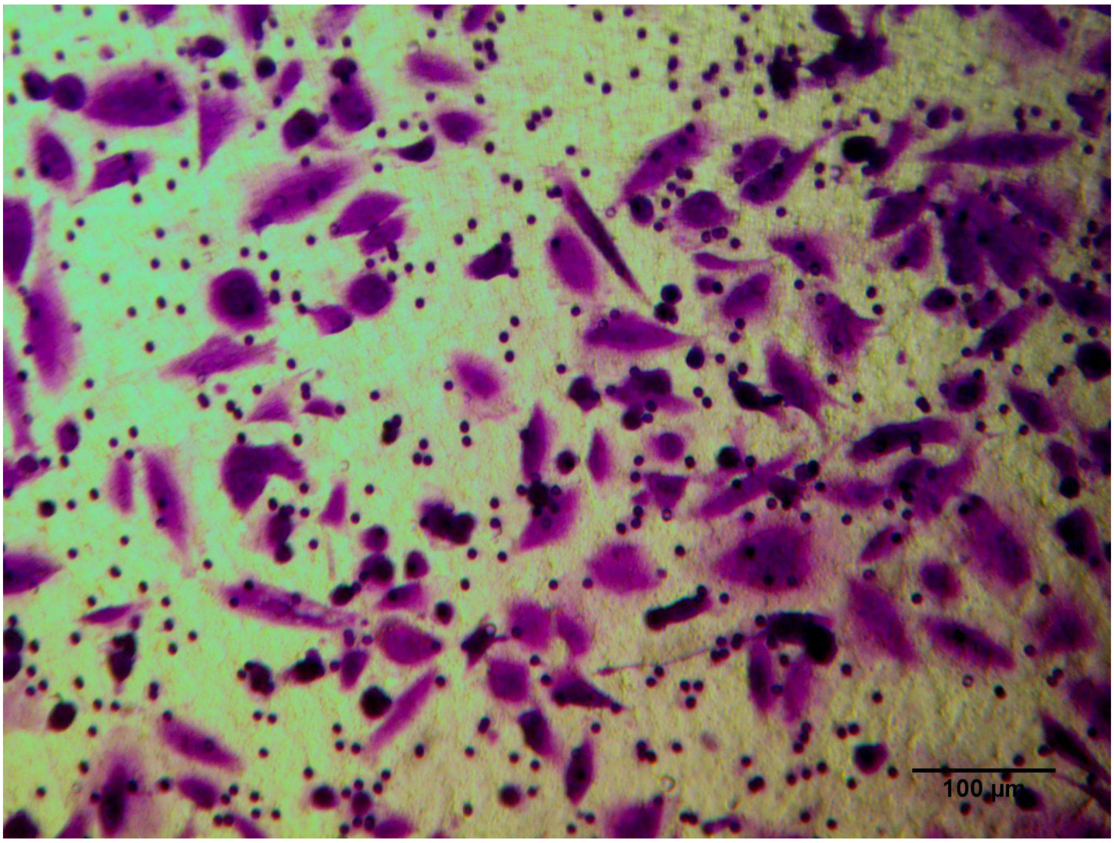

Transwell-SCC-4-NC(2)

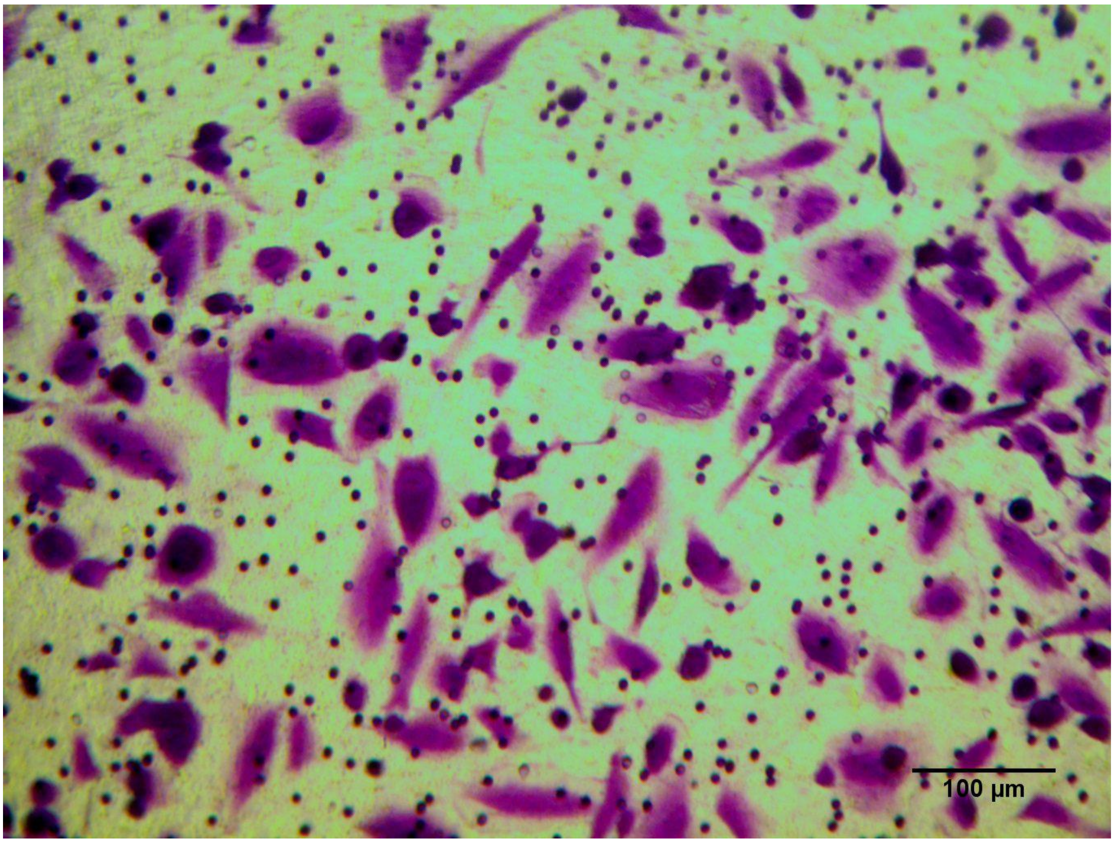

Transwell-SCC-4-NC(3)

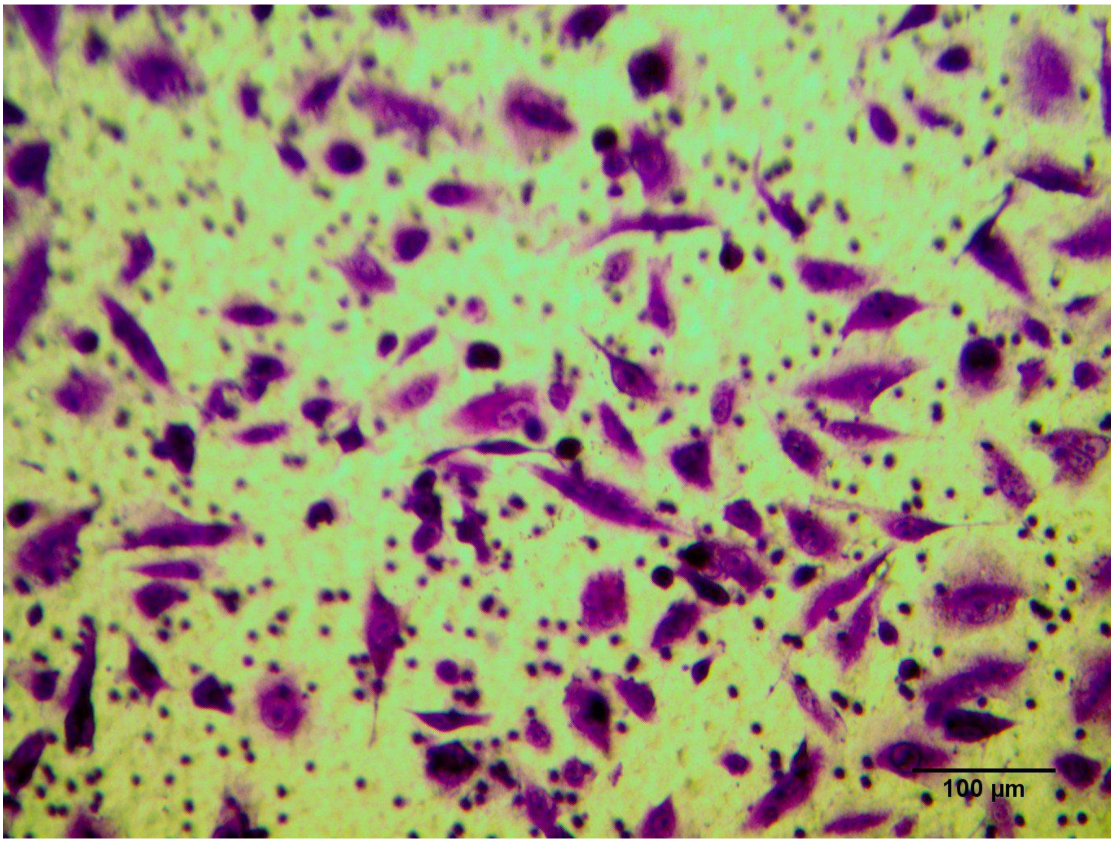

Transwell-SCC-4-NC(4)

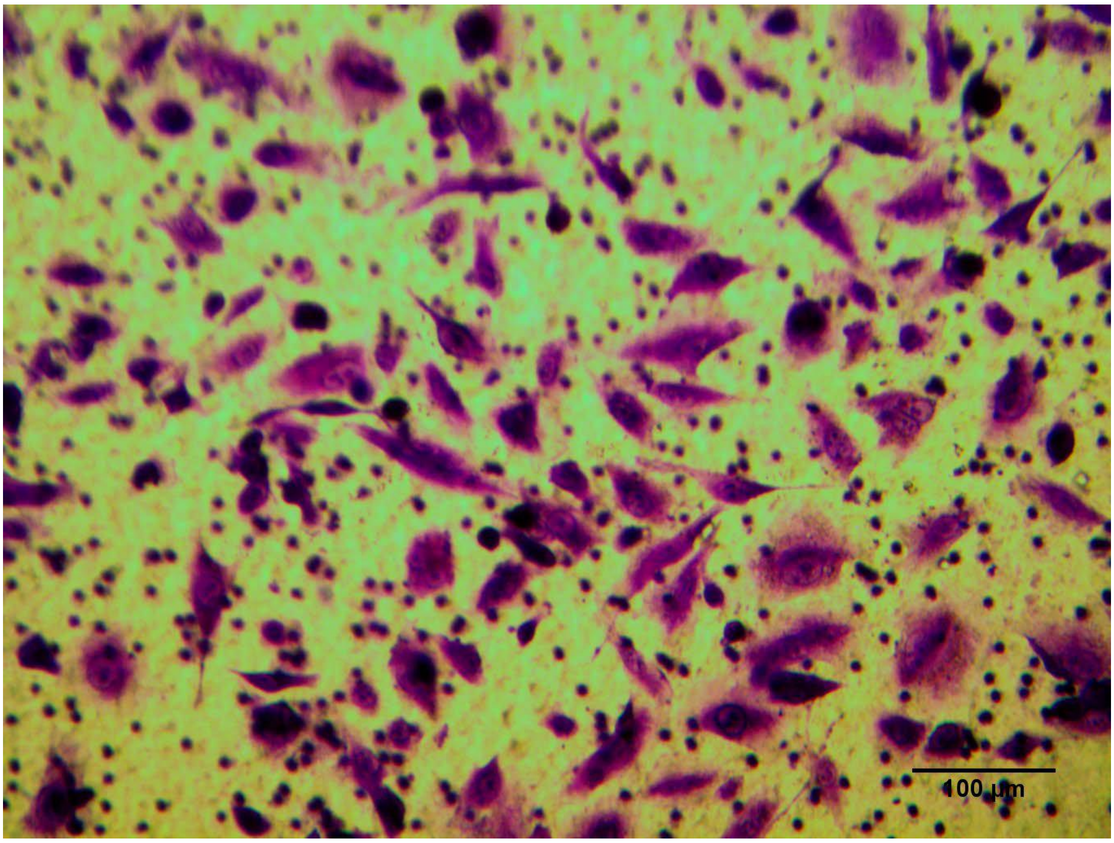

Transwell-SCC-4-NC(5)

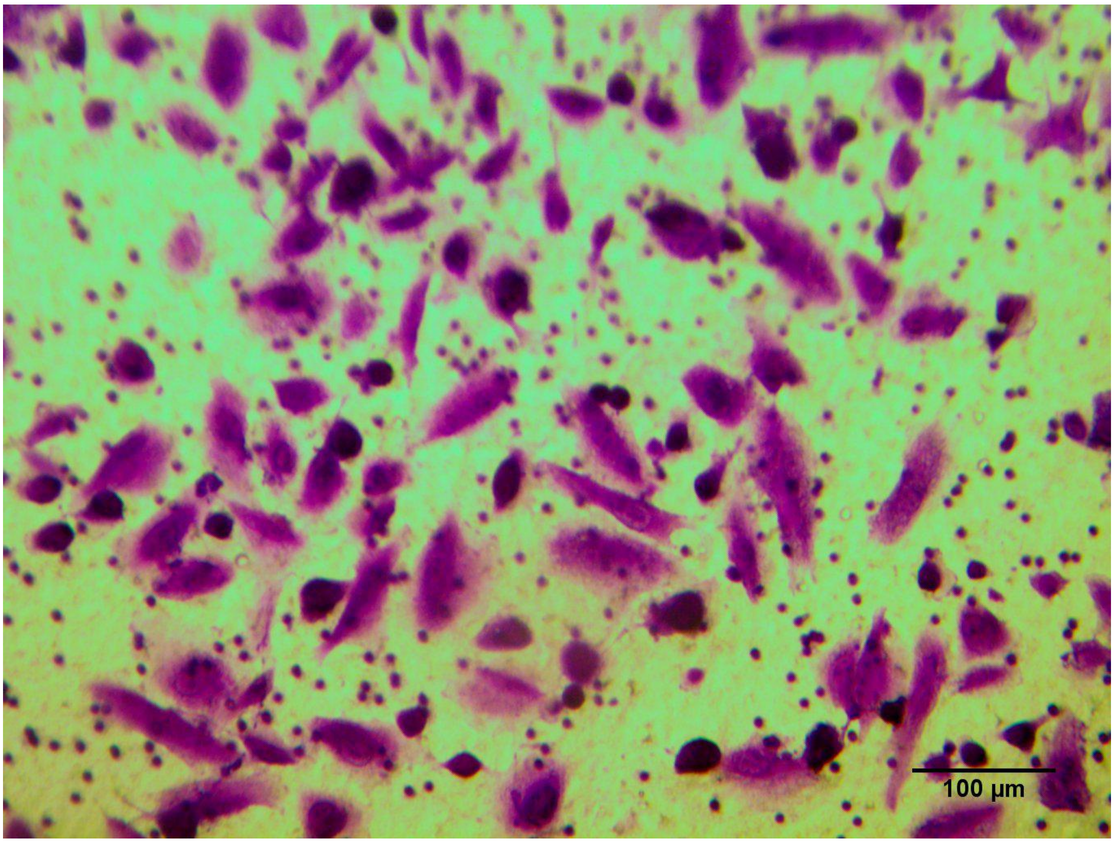

Transwell-SCC-4-siRNA(1)

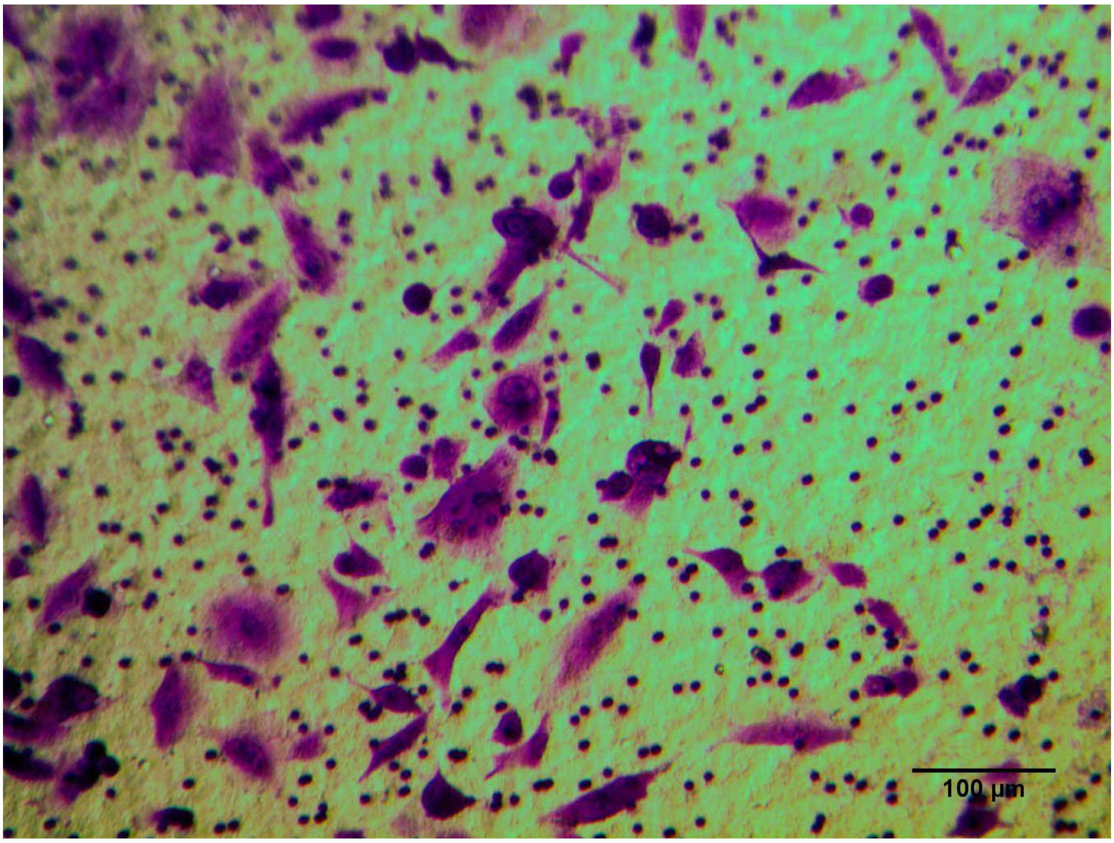

Transwell-SCC-4-siRNA(2)

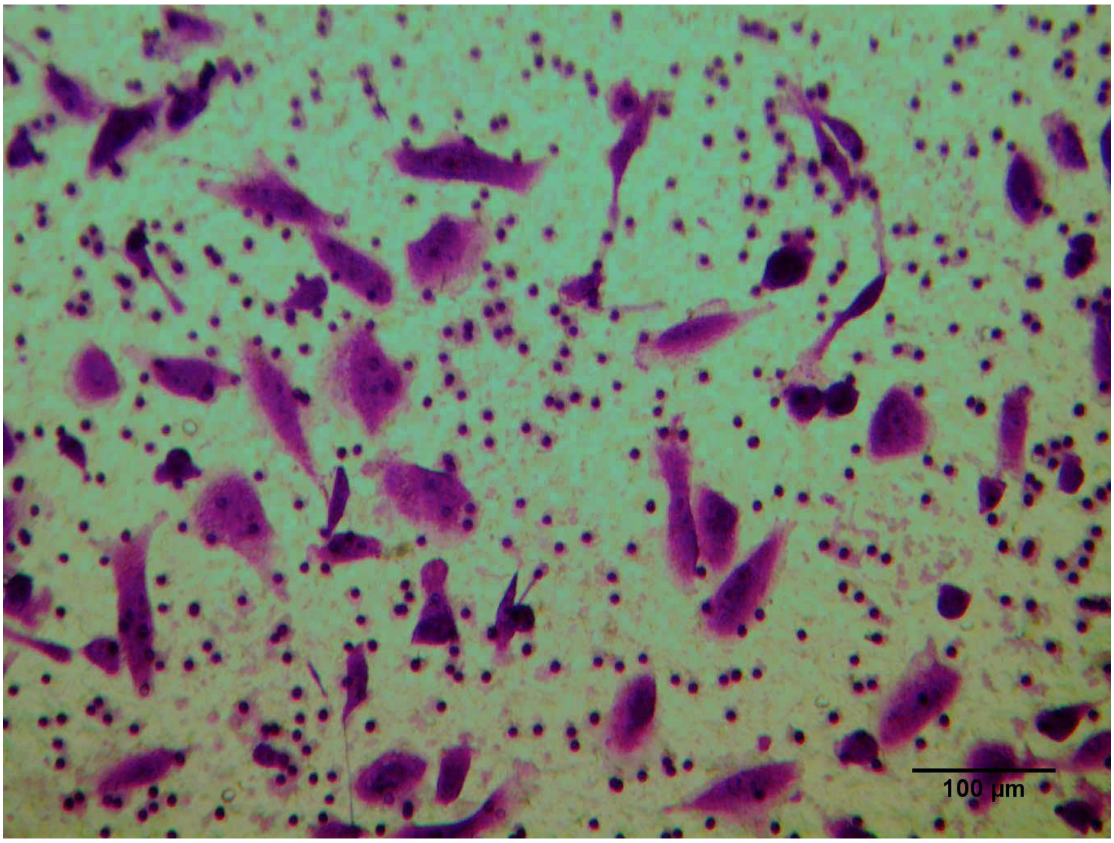

Transwell-SCC-4-siRNA(3)

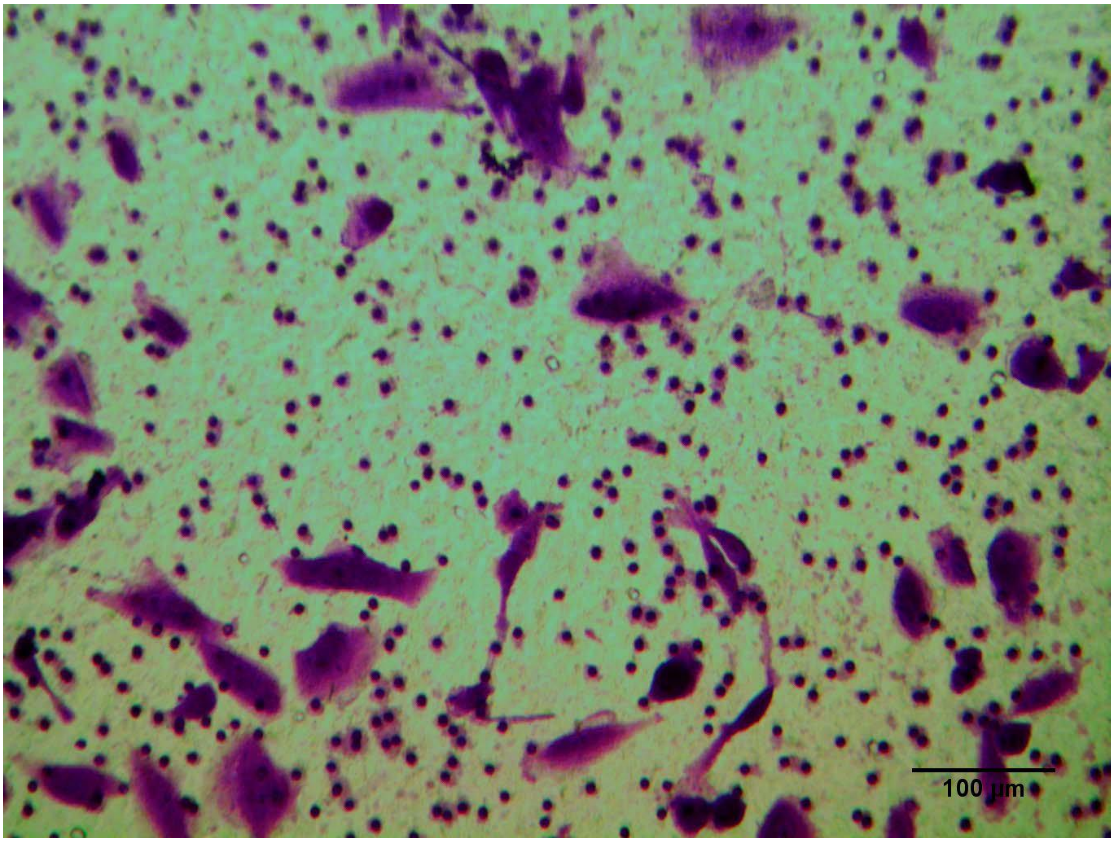

Transwell-SCC-4-siRNA(4)

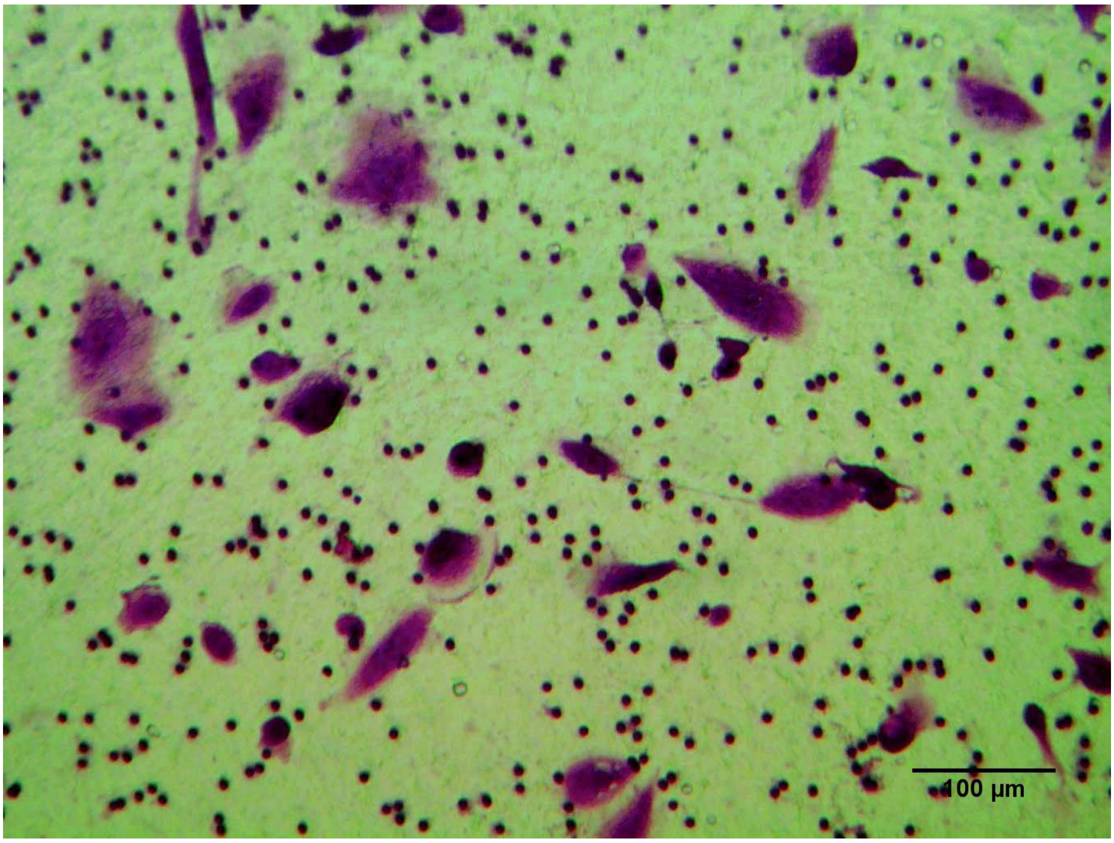

Transwell-SCC-4-siRNA(5)

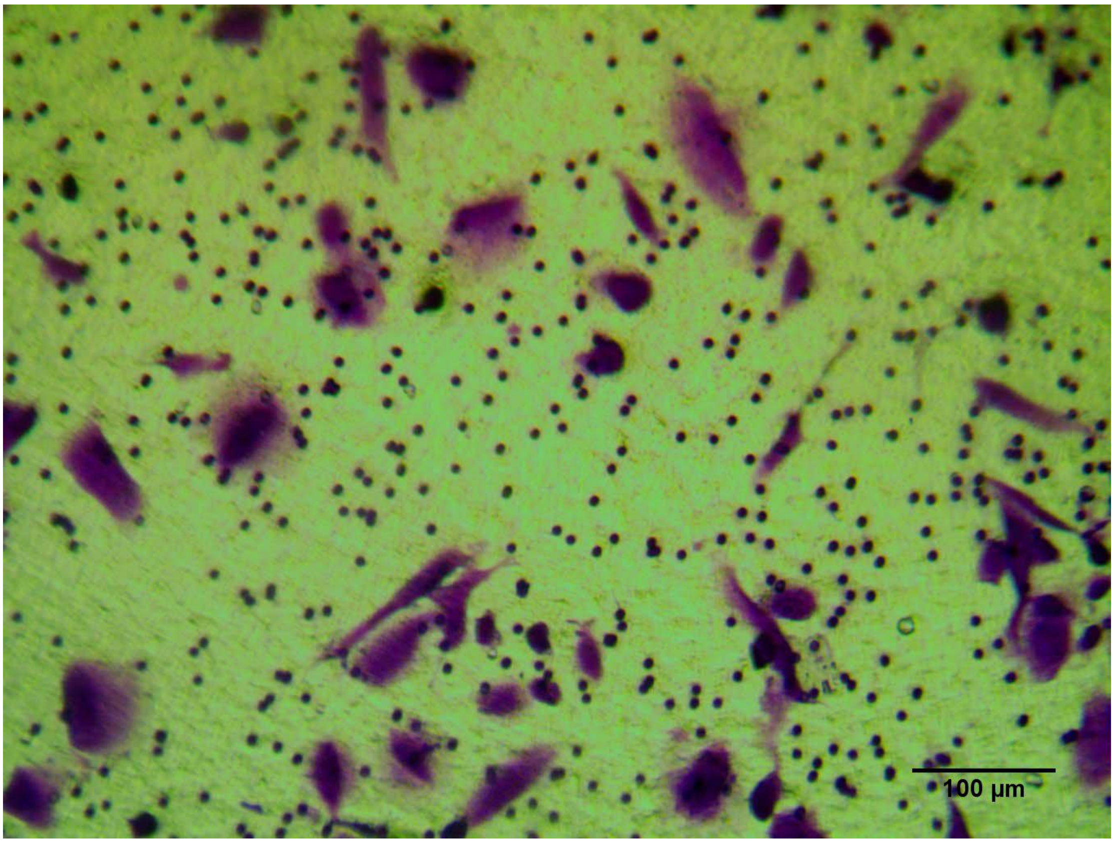

Western blot

Fig.3 Original western blot1

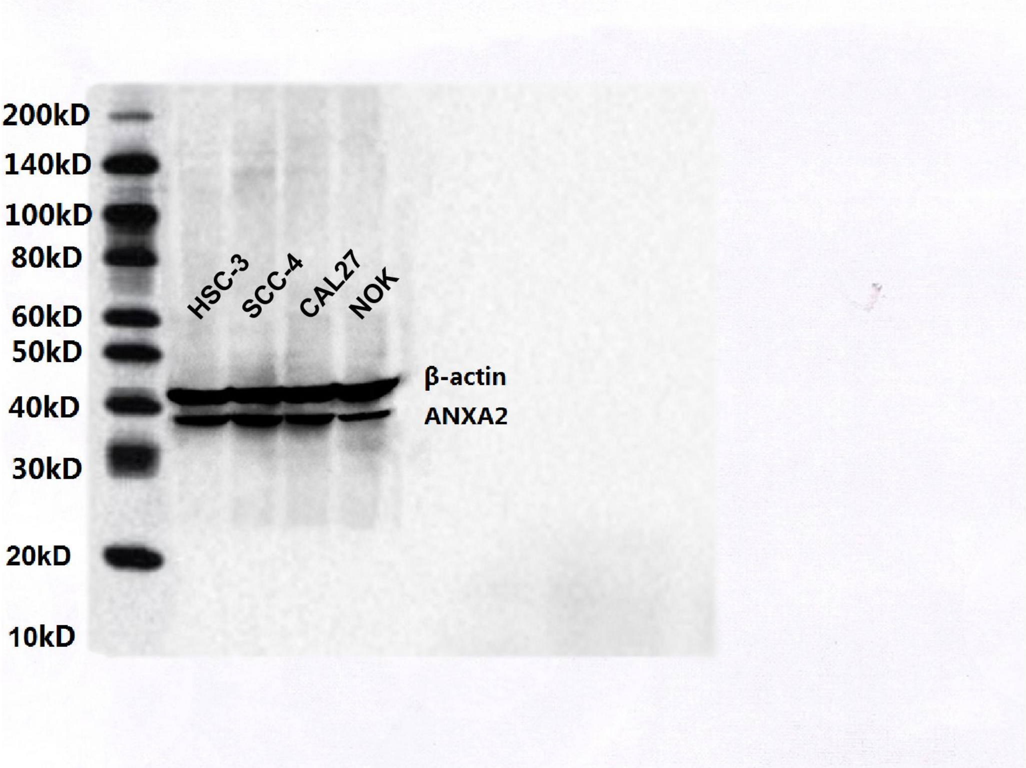

Fig.4 Original western blot1

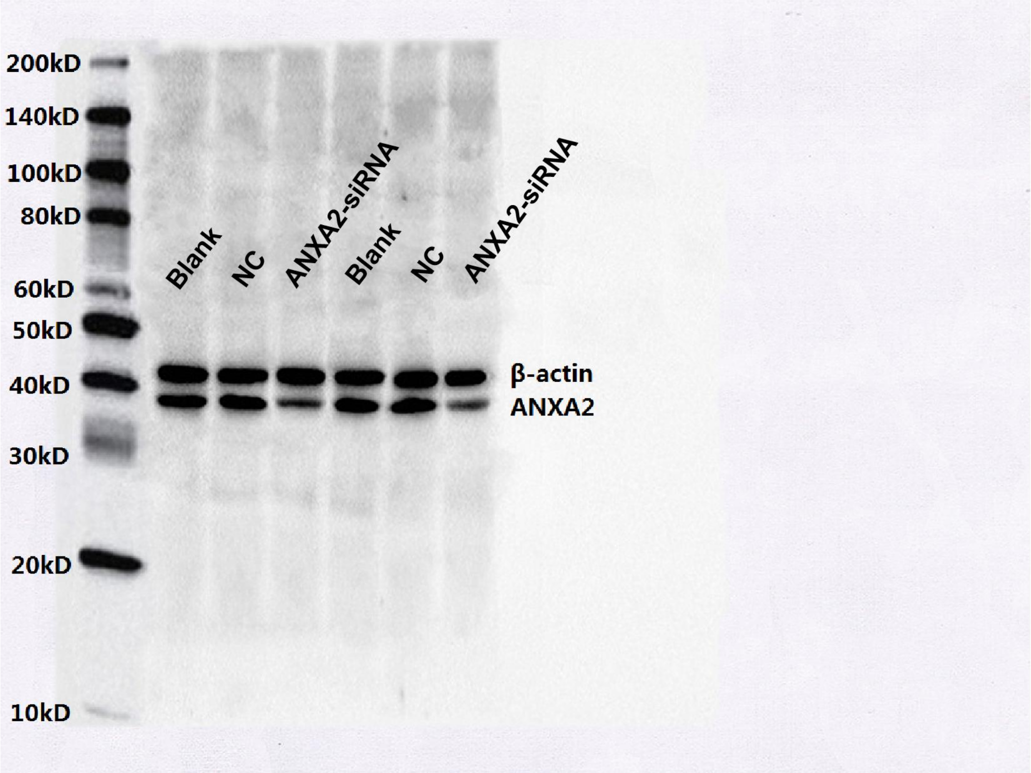

Original Data

No.	TNM stage	differentiation	LNM	staining intensity of IHC	
				OSCC Tissue	Noncancer tissue
1	Ⅰ	L	Y	2	1
2	Ⅰ	L	N	1	0
3	Ⅱ	M	Y	3	2
4	Ⅰ	M	Y	2	1
5	Ⅰ	L	Y	2	1
6	Ⅰ	H	N	1	0
7	Ⅱ	M	Y	3	2
8	Ⅱ	M	Y	3	1
9	Ⅲ	H	N	2	0
10	Ⅳ	H	Y	3	2
11	Ⅰ	M	N	1	0
12	Ⅲ	H	N	1	0
13	Ⅲ	M	N	2	1
14	Ⅰ	L	N	0	0
15	Ⅲ	M	N	2	1
16	Ⅰ	L	Y	2	0
17	Ⅳ	H	N	3	2
18	Ⅲ	H	N	3	2
19	Ⅲ	M	N	2	1
20	Ⅱ	L	N	3	2
21	Ⅲ	M	N	2	1
22	Ⅲ	H	N	1	0
23	Ⅳ	H	N	3	1
24	Ⅲ	M	N	2	1
25	Ⅰ	L	Y	2	0
26	Ⅰ	L	Y	2	1
27	Ⅱ	M	N	3	2

28	II	M	N	2	1
29	I	L	Y	2	1
30	I	L	Y	2	1
31	I	H	N	1	0
32	II	M	N	3	2
33	II	M	N	3	2
34	I	L	Y	2	0
35	II	M	N	3	2
36	I	L	Y	2	0
37	II	H	N	1	0
38	III	H	N	3	1
39	III	H	N	3	1
40	I	L	N	0	0
41	III	H	N	1	0
42	IV	H	N	3	2
43	III	H	N	2	0
44	III	M	N	2	0
45	III	H	N	3	1
46	III	H	N	3	1
47	I	L	Y	2	0
48	II	H	N	1	0
49	II	M	N	3	2
50	I	M	Y	2	1
51	I	M	Y	2	1
52	II	M	N	3	1
53	II	M	N	3	2
54	I	L	Y	2	1
55	II	M	N	3	2
56	II	L	N	2	1
57	III	M	N	2	1

58	Ⅳ	H	N	3	2
59	Ⅱ	H	N	1	0
60	Ⅲ	M	N	2	1
61	Ⅱ	L	N	0	0
62	Ⅲ	M	N	2	1
63	Ⅲ	H	N	3	2
64	Ⅰ	L	Y	2	1
65	Ⅰ	L	Y	3	1
66	Ⅰ	H	N	1	1
67	Ⅱ	M	N	3	1
68	Ⅰ	M	Y	3	0
69	Ⅰ	L	N	1	0
70	Ⅲ	M	N	2	1
71	Ⅲ	H	N	3	2
72	Ⅲ	M	N	2	0
73	Ⅳ	H	N	3	1
74	Ⅰ	M	N	1	0
75	Ⅲ	M	N	2	0
76	Ⅲ	L	N	2	0
77	Ⅱ	H	N	1	1
78	Ⅲ	M	N	2	0
79	Ⅲ	H	N	3	2
80	Ⅲ	M	N	2	0
81	Ⅲ	H	N	3	1
82	Ⅱ	H	N	1	0
83	Ⅱ	M	N	3	2
84	Ⅱ	L	Y	2	0
85	Ⅰ	L	Y	2	1
86	Ⅱ	M	N	1	0
87	Ⅰ	L	Y	2	1

88	Ⅰ	L	Y	2	0
89	Ⅱ	M	N	3	1
90	Ⅰ	L	N	2	0
91	Ⅲ	H	N	2	0
92	Ⅱ	H	N	1	1
93	Ⅲ	M	N	2	0
94	Ⅳ	H	N	3	1
95	Ⅲ	H	N	2	1
96	Ⅱ	H	N	1	0
97	Ⅲ	M	N	2	0
98	Ⅲ	M	N	2	0
99	Ⅰ	L	N	0	0
100	Ⅱ	L	N	1	0
101	Ⅲ	H	N	1	1
102	Ⅲ	H	N	3	2
103	Ⅲ	M	N	2	0
104	Ⅲ	H	N	3	2
105	Ⅲ	M	N	2	0
106	Ⅲ	H	N	3	2
107	Ⅲ	H	N	1	1
108	Ⅲ	M	N	2	1
109	Ⅱ	M	N	3	2
110	Ⅰ	L	Y	2	1
111	Ⅲ	M	Y	1	1
112	Ⅲ	H	N	1	1
113	Ⅱ	M	N	3	2
114	Ⅲ	H	N	3	2
115	Ⅱ	L	N	2	0
116	Ⅱ	H	N	3	2
117	Ⅱ	M	N	3	2

118	II	L	Y	2	0
119	III	H	N	2	1
120	IV	H	N	3	2
121	III	H	N	3	2
122	II	L	N	3	2
123	II	L	N	2	0
124	IV	H	Y	1	1
Note		L=low<br>M=moderately<br>H=high	N=no<br>Y=yes	0~1=negative<br>2~3=positive	0~1=negative<br>2~3=positive
